# An emerging trend in Novel Psychoactive Substances (NPSs): designer THC

**DOI:** 10.1186/s42238-024-00226-y

**Published:** 2024-05-03

**Authors:** Cristian Caprari, Elena Ferri, Maria Angela Vandelli, Cinzia Citti, Giuseppe Cannazza

**Affiliations:** 1 Clinical and Experimental Medicine PhD Program, University of Modena and Reggio Emilia, Modena, 41125 Italy; 2 Department of Life Sciences, University of Modena and Reggio Emilia, Via G. Campi 103, Modena, 41125 Italy; 3 Institute of Nanotechnology of the National Council of Research – CNR NANOTEC, Campus Ecotekne, Via Monteroni, Lecce, 73100 Italy

**Keywords:** THC, HHC, NPS, Synthetic cannabinoids, Designer drugs

## Abstract

Since its discovery as one of the main components of cannabis and its affinity towards the cannabinoid receptor CB1, serving as a means to exert its psychoactivity, Δ^9^-tetrahydrocannabinol (Δ^9^-THC) has inspired medicinal chemists throughout history to create more potent derivatives. Initially, the goal was to synthesize chemical probes for investigating the molecular mechanisms behind the pharmacology of Δ^9^-THC and finding potential medical applications. The unintended consequence of this noble intent has been the proliferation of these compounds for recreational use. This review comprehensively covers the most exhaustive number of THC-like cannabinoids circulating on the recreational market. It provides information on the chemistry, synthesis, pharmacology, analytical assessment, and experiences related to the psychoactive effects reported by recreational users on online forums. Some of these compounds can be found in natural cannabis, albeit in trace amounts, while others are entirely artificial. Moreover, to circumvent legal issues, many manufacturers resort to semi-synthetic processes starting from legal products extracted from hemp, such as cannabidiol (CBD). Despite the aim to encompass all known THC-like molecules, new species emerge on the drug users’ pipeline each month. Beyond posing a significantly high public health risk due to unpredictable and unknown side effects, scientific research consistently lags behind the rapidly evolving recreational market.

## Introduction

“In 1962 I started research on cannabis with the expectation that it would be a minor project to be completed within 6 months. Forty years hence, I am still working in the field, having published more than 250 articles on it” (Mechoulam 2006). Indeed, echoing Mechoulam’s sentiments, cannabis continues to capture interest across various sectors of society, ranging from scientific and industrial communities to clinics and clandestine laboratories. The primary driver of this widespread attention lies in the plant’s ability to produce a distinctive class of terpenophenolic compounds known as phytocannabinoids. Among the over 150 identified phytocannabinoids, the most extensively studied is unquestionably Δ^9^-tetrahydrocannabinol (Δ^9^-THC), first isolated by R. Mechoulam in the early 1960s (Gaoni and Mechoulam 1964). Following its isolation and structural elucidation, it was discovered that this compound induced ataxia in dogs and a state of drowsiness in monkeys (Grunfeld and Edery 1969; Mechoulam et al. 1970). Later studies concluded that Δ^9^-THC is the primary psychoactive component of cannabis responsible for the “high” effects achieved by stimulating specific receptors in the central nervous system (CNS). The effects produced by Δ^9^-THC on the CNS are diverse, including CNS depression, ataxia, psychoactive effects, hypothermia, analgesia, cardiovascular effects, and more. At a molecular level, these effects are known to alter neurotransmitter functionality, enzyme activity, prostaglandin synthesis, membrane perturbation, and so forth. Over the years, research has attributed this so-called “cannabis-like” or “THC-like” effect to the activation of the endocannabinoid receptor 1 (CB1R) in the CNS, involving several structural requirements to generate a cascade of physiological effects.

The first structure-activity relationship (SAR) studies on THC were carried out by Mechoulam and Edery in the early 1970s primarily focusing on investigations involving rhesus monkeys and dogs (Edery et al. 1972). These studies revealed that the essential prerequisites for activity were the benzopyran ring system and the aliphatic side chain. The alicyclic ring attached to the benzopyran system, on the other hand, could be substituted by a heterocycle with a broad range of substituents without a loss of activity. Significant emphasis was placed on the aliphatic side chain, which needed to be no shorter than 3 carbon atoms. Moreover, an increasing number of carbons in the side chain was found to confer higher potency (Mechoulam et al. 1987). Lastly, hydroxylation at certain positions, such as the methyl group on the alicyclic ring (11-OH-THC) or the aliphatic side chain (3”-OH-Δ^9^-THC), maintained or even enhanced activity (Mechoulam et al. 1987).

Building on this understanding, numerous variations have been introduced to the lead compound Δ^9^-THC with the aim of enhancing the affinity for CB1R and, consequently, the cannabimimetic activity. A considerable number of novel cannabinoids bearing the THC scaffold were synthesized initially with the goal of developing new therapeutic drugs with improved pharmacological activity. These compounds are emerging as Novel Psychoactive Substances (NPSs), which are defined by the United Nations Office on Drugs and Crime (UNODC) as “substances of abuse, either in pure form or in preparation, that are not controlled by the United Nations drug conventions, but which may pose a public health threat comparable to that posed by substances listed in these conventions”(UNODC. 2024). These substances are not necessarily “new” in terms of discovery, as many of them have been known for decades, but rather have recently entered the recreational market (UNODC 2024).

### Classification of THC-like NPS

In this scenario two big classes of compounds can be identified. One class of these THC-like cannabinoids features substitutions on the central core of the lead compound (benzopyran ring system, alicyclic ring, and aliphatic side chain), rendering the chemical structure entirely artificial and not traceable to a naturally occurring cannabinoid. Therefore, these compounds could be referred to as “non-natural (THC-like) cannabinoids” (NNCs). The other class includes compounds found in nature, albeit in trace amounts, but not obtainable through simple extraction like the lead compound Δ^9^-THC. Hence, the most suitable term to be as inclusive as possible is probably “pseudo-natural (THC-like) cannabinoids” (PNCs).

Compounds belonging to both classes can be obtained by semi-synthesis (SS) when the precursor, either Δ^9^-THC or cannabidiol (CBD), is easily accessible through extraction from plant genetics expressing such compounds in high amounts. Once extracted, the precursor undergoes chemical synthesis to obtain highly potent CB1R agonists (either SSPNCs or SSNNCs). Alternatively, THC-like compounds can be prepared by total synthesis when neither Δ^9^-THC nor CBD can be used as starting material (either SPNCs or SNNCs). Such terminology has been proposed by the authors and is not officially reported elsewhere. Rossheim et al. addressed the issue of terminology proposing the term *derived psychoactive cannabis products* (Rossheim et al. 2023), which is appropriate but unable to encompass all species currently available in the recreational market. For instance, the adjective *psychoactive* excludes all non-psychoactive cannabinoids like CBG or CBD, but the term *derived* does not include THCA, which is totally natural and sold as crystals under the name “THCA diamonds”. For this reason, we believe it is not possible to cover such a broad range of compounds with a single terminology and propose to use different groups (or classes) to indicate natural occurrence and type of synthesis.

#### Non-natural cannabinoids (NNCs)

Between the end of 1980s and the beginning of 2000s, Mechoulam’s SAR investigations led to the fruitful development of several synthetic THC analogues with promising therapeutic properties (Mechoulam 2006). Nevertheless, all efforts to take at least a few of these molecules to clinical use have failed (Diao and Huestis 2019). The reason for this failure, besides the lack of a reliable animal model generating quantifiable effects which can be extrapolated to humans, has been supposed to lie in the challenge of separating the therapeutic effects from the psychoactive and the other undesirable CNS side effects (Razdan 1987). Indeed, the quest for increasingly potent molecules led to a misuse of NNCs and a shift of the target end-users from patients to drug consumers with consequent severe intoxications and deaths reported (EMCDDA 2021; Logan et al. 2017). The fatal cases were attributed to an overdose of CB1R/2R agonists, highlighting the significant difference between full agonists and partial agonists (such as Δ^9^-THC) (Sholler et al. 2020). At present, the class of NNCs, together with that of synthetic cannabinoids (SCs), represents the largest class of drugs detected through the EU Early Warning System with a total of 209 substances notified between 2008 and 2019 (EMCDDA 2021). SCs present a structure that cannot be traced back to that of Δ^9^-THC and are indicated with several different numbers associated to acronyms like “JWH” or “AM” just to make an example (Alves et al. 2020). Such SCs are beyond the scope of the present review, thus they will not be included in the discussion.

Since their initial spread, NNCs have grown in number and evolved in structural diversity to circumvent forensic detection and drug scheduling. Moreover, compared to their lead compound, they exhibit a different pharmacokinetic profile in terms of duration of the effects, plasma half-life, and metabolites and degradants generated. This scenario complicates the monitoring and detection as well as the real estimation of cannabinoid prevalence on the market, thus representing a major challenge for policymakers (Alves et al. 2020).

In addition to the synthetically obtained compounds, NNCs also include compounds that can be prepared starting from the lead compound THC or its isomer CBD. The choice to use the latter as a precursor turned out to be an alternative strategy to evade the legal issues related to the extraction of THC from illegal drug-type (high-THC) cannabis varieties. With the revival of industrial hemp or “low-THC cannabis” in both Europe and the USA, there has been a burst in the cultivation of cannabis for industrial purposes (hemp), encouraging the marketing of hemp products. Industrial (fibre-type) hemp comprises cannabis varieties characterized by higher concentrations of the well-known non-narcotic CBD than those of the psychoactive THC (< 0.3% w/w). As a result, there has been a rapid development of high-CBD varieties, which in recent years led to an oversupply of this phytocannabinoid, thus prompting the market to shift towards other cannabinoids. This was also encouraged by the rediscovery of the ancient knowledge of the possible conversion of CBD into other THC-like molecules (Järbe et al. 1986; Gaoni and Mechoulam et al. 1966), which have been called semi-synthetic cannabinoids (SSCs). In this context, NNSSCs include for example the acetylated version of THC, namely Δ^9^-THC-acetate (AcO-Δ^9^-THC, sometimes indicated as either THCOa or Δ^9^-THC-O) (Valentine 1996).

#### Pseudo-natural cannabinoids (PNCs)

The class of PNCs embraces structures that strongly resemble that of Δ^9^-THC. An outstanding example of this class is represented by the regioisomer Δ^8^-THC, which has a cannabimimetic activity comparable to or slightly lower than that of Δ^9^-THC (Razdan 1987). Δ^8^-THC is found in traces in the plant, particularly in aged cannabis, and derives from the shift of the double bond from the Δ^9^ to the more thermodynamically stable position Δ^8^ (Dalzell et al. 1981). However, although Δ^8^-THC can be easily prepared from CBD through an acid-catalysed cyclization as first reported by Gaoni and Mechoulam (Gaoni et al. 1966), the product Δ^8^-THC is a Schedule I controlled substance included in the 1971 Convention on Psychotropic Substances (United 1971), thus not legally marketable. In order to circumvent the legal issue, both Δ^8^-THC and Δ^9^-THC can be converted into their hydrogenated version, (9*R*)-hexahydrocannabinol ((9*R*)-HHC) and (9*S*)-HHC respectively (Russo et al. 2023). The phenomenon of HHC, the first SSPNC, was initially thought to be confined to the USA (EMCDDA 2023). However, by mid-2022, the uncontrolled sales of HHC products of largely unknown purity crossed the borders and started to spread all over the European territories as well (EMCDDA 2022). While HHC would fall in the semi-synthetic subgroup of the PNCs as this molecule has been identified in traces in cannabis plant material as a degradation product of Δ^9^-THC (Hanuš et al. 2016), a new trend regards the marketing of its acetylated version HHC-acetate (AcO-HHC, sometimes indicated as either HHCOa or HHC-O) (Ujváry 2023), which instead would belong to the SSNNCs.

Other naturally occurring cannabinoids that cannot be prepared from either CBD or Δ^9^-THC, thus requiring a full synthetic process are represented by the recently discovered Δ^9^-tetrahydrocannabutol (Δ^9^-THCB) (Linciano et al. 2020a), Δ^9^-tetrahydrocannabiphorol (Δ^9^-THCP) (Citti et al. 2019), and Δ^9^-tetrahydrocannabihexol (Δ^9^-THCH) (Linciano et al. 2020b), which are the butyl, heptyl, and hexyl homologs of Δ^9^-THC, respectively. Δ^9^-THCP has drawn particular attention from both the scientific and industrial communities to the point that this compound is now being sold in many products including tinctures, distillates, vape cartridges, and gummies (Schmidt 2023). As a result of merging the characteristics of Δ^9^-THCP and HHC, an heptyl homolog of HHC called hexahydrocannabiphorol (HHCP) has appeared on the market and can be easily purchased from online vendors.

Ancient knowledge of synthetic methodologies and biological studies and more recent discoveries of new THC-like compounds have merged to provide a novel experience for the drug users. Unfortunately, biological studies lack for most compounds and many side effects are unknown. In this context, scientific research is always one step behind the introduction of new drugs of abuse and the outcomes for the social health and safety are unpredictable. Nonetheless, the lead compound Δ^9^-THC remains a valuable and promising starting point for therapeutic drug development and treatment of pathologies unresponsive to conventional medications.

All THC-like NPSs are reported in Table 1 with structure, natural occurrence, type of synthesis, identification methodology, in vitro pharmacological details and effects of recreational use.

**Table 1:** THC-like cannabinoids in the recreational market: natural occurrence, manufacturing process, structure, methods used for analytical assessment, CAS registry number, in vitro evaluation of CB1R binding affinity (K_i_) in relation to the K_i_ of the reference Δ^9^-THC and experienced effects after recreational use

Natural occurrence	Manufacturing process	Structure^a^	Analytical assessment	CAS RN^b^	CB1R affinity (K_i_) (nM) (Ref.)	Reference K_i_ for Δ^9^-THC (nM)	Recreational effects
Naturally occurring in traces (PNCs)	Semi-synthesis (SSPNCs)		GC-MS with derivatization (Nahar et al. 2020) HPLC-HRMS (Nahar et al. 2020) HPLC-DAD (SCLabs 2022; Inc. SAS 2023) HPLC-UV (Analytical 2021)	23978-85-0	3100 (Zagzoog et al. 2020)	50	Vaping or dabbing are the recommend ways to consume them in progressively increasing doses (TheChronicBeaver 2021; Bortolazzo n.d.) The best experience in terms of “high” effects and taste is achieved by blending them with terpenes or cannabis (reddit.com. 2021)
			HPLC-UV (Citti et al. 2020; –Song et al. 2022) HPLC-HRMS (Kiselak et al. 2020; Pellati et al. 2018) GC-MS (Sams 2022)	5957-75-5	45 ± 12 (Martin et al. 1999)	41 ± 1.7	Milder psychoactive and side effects compared to Δ^9^-THC (Schmidt 2023)
			NP-HPLC-UV (Williams et al. 2021) Chiral HPLC-UV (Umstead 2021) GC-MS (Ciolino et al. 2021) 2D-LC-MS/MS (Chan-Hosokawa et al. 2021)	95543-62-7 (9*R*) 95588-87-7 (9*S*)	-	-	Uplifting and energizing effects (Schmidt 2023) Enhancement of motivation, creativity, and cognitive function (Schmidt 2023) Less potent than Δ^8^-THC (reddit.com. 2020)
			NP-HPLC-UV (Williams et al. 2021) Chiral HPLC-UV (Umstead 2021) GC-MS (Ciolino et al. 2021) 2D-LC-MS/MS (Chan-Hosokawa et al. 2021)	95720-01-7 (9*R*) 95720-02-8 (9*S*)	5 ± 2 (Rosati et al. 2014)	22 ± 13	Milder effects than Δ^9^-THC (Schmidt 2022) Uplifting and energizing effects Heightened sense of mental clarity and focus without paranoia and anxiety (Schmidt 2022)
			GC-MS, GC-FID, NMR, FT-IR (Friedrich-Fiechtl and Spiteller 1975; Kettenes-van den Bosch and Salemink 1977) HPLC-MS (Lewis et al. 2017)	56154-60-0^*c*^	-	-	Supposed similar properties as Δ^9^-THC (Hewett 2022; What is 10-Oxo-Δ6a(10a)-tetrahydrocannabinol (OTHC)? 2023)
			NP-HPLC-UV (Banijamali and Makriyannis 1987) RP-HPLC-UV (Krepich et al. 2020; Franklin and Wilcox 2019) RP-HPLC-DAD (Ciolino et al. 2021)	27179-28-8	(IC_50_) 334 (Compton et al. 1991)	(IC_50_) 218	“Good spacey feeling” and slow reaction time, though keeping a clear head sensation (reddit.com. 2023)
			GC-MS (Andrenyak et al. 2017; , Purschke et al. 2016) LC-MS/MS (Reinstadler et al. 2023; Simões et al. 2011)	36557-05-8	0.37 (Zagzoog et al. 2022)	35	Quick onset and a strength close to THC, along with an accelerated heartbeat and racing thoughts (reddit.com. 2023)
			GC-MS (Vree et al. 1972; Vree et al. 1973) HPLC-UV, NMR, TLC (Stothard et al. 2023)	6692-85-9 (racemic) 36403-90-4 (9*R*) 36403-91-5 (9*S*)	19 (Ujváry 2023)	27	Effects comparable to those of Δ^9^-THC (sedation and relaxation, euphoric high and a smooth, calming experiencel) and sporadic side effects like psychosis, uncontrolled tremors, etc. (reddit.com 2023; Schmidt 2023; reddit.com 2023).
			GC-MS, LC-MS, NMR (Cayman 2023; Harvey 1981)	73648-83-6 (9*R*,10*S*) 60948-21-2 (9*S*,10*R*)	-	-	Effects similar to those of HHC itself, although a bit shorter in duration (reddit.com. 2023)
			-	2041284-40-4	-	-	Not used as such but after decarboxylation into HHC
			-	60491-22-7	-	-	Expected to have the same effects as Δ^9^-THC, but more reactive to liver metabolism (Jagannathan 2020)
	Synthesis (SPNCs)		HPLC-HRMS (Linciano et al. 2020a)	60008-00-6	15 (Linciano et al. 2020a)	41	Effects either comparable or higher to those of Δ^9^-THC (Brandrup 2023; Future4200. 1807) Quick sensations of euphoria, mental energy and uplift, bodily relaxation and calmness (Brandrup 2023)
			HPLC-HRMS (Linciano et al. 2020b)	36482-24-3	-	-	The psychoactive effect lasts longer than that of Δ^9^-THC (reddit.com, 2023)
			ELISA (Moody et al. 2022) HPLC-HRMS (Citti et al. 2019)	54763-99-4	1.2 (Citti et al. 2019)	40	More potent than Δ^9^-THC Effects could last up to 24 h or more (reddit.com. 2022)
			-	1349000-38-9^*c*^	-	-	Fast onset and decrease of the effects (edibles) (TSM. 2023)
			-	1348837-48-8^*c*^	-	-	Vaping HHCH distillates gives a mild sensation, much lower than the corresponding THC hexyl counterpart THCH (reddit.com. 2023) HHCH edibles produce a high psychoactive effect (reddit.com. 2023)
			GC-MS, NMR (EMCDDA. 2023)	2891843-81-3^*c*^	-	-	Unprecedented euphoria, intense uplifting effects and mood boost (Schmidt 2023)
Unnatural (NNCs)	Semi-synthesis (SSNNCs)		ELISA (Moody et al. 2022) DART-QToF, GC-MS, LC-MS/MS (Holt et al. 2022)	23132-17-4	-	-	Stronger and delayed effects with respect to Δ^8^-THC and Δ^9^-THC, sometimes with some psychedelic effects like increased time dilation, deeply internalized self-contemplation and complete focus on only one thing at a time (reddit.com. 2021)
			DART-QToF, GC-MS and LC-MS/MS (Holt et al. 2022)	-	-	-	Effects 1.5 times higher than HHC (What is HHC-O? The Complete Guide 2022) Sedation and relaxation, removal of overwhelming thoughts, more relaxed sleep, blissfulness, openness to new sensations and ideas (binoid 2022; Heredia 2022) Later onset of the effects compared to other cannabinoids (reddit.com. 2023) Episodes of panic attacks after consuming edibles with a sort of chronic depressive mood lasting for a few weeks afterwards (reddit.com. 2023)
			GC-MS (YLA 2023; Sams 2023)	36403-93-7 (8*S*,9*R*) 42793-11-3 (8*R*,9*S*)	-	-	Quicker onset of the effects compared to HHC but the physical and mental experience is basically the same (reddit.com. 2023)
	Synthesis (SNNCs)		GC/MS (DEA 2019) *Quantisal*device (Coulter et al. 2011)	112830-95-2	0.23 ± 0.03 (Howlett et al. 1990)	1.6	Higher potency compared to Δ^9^-THC, high safety (reddit.com. 2023), and long-lasting effects (Bluelight 2018) Low doses provide the desired “high” effects in very short time, high doses can produce dissociative and psychedelic effects, and medium doses have good analgesic properties (Bluelight 2016)
			-	2829292-82-0	-	-	Longer lasting and more euphoric effects compared to THCP, but also a later onset (up to 30–45 min) (Bluelight 2023; Black et al. 2023) Euphoria, relaxation, intoxication, or drowsiness (Canatura 2023) Anxiety, drowsiness, dry mouth, red eyes, and paranoia (Black et al. 2023)
			-	2829292-83-1^*c*^	-	-	Strong and long-lasting effects, but slow onset of the effects (reddit.com. 2023; Thomas 2023) Relaxation, intense euphoria, pain relief, warm body vibrations, and creative inspiration (DottorCanapa 2023)
			HPLC (reddit.com. 2023)	2552798-63-5	8.5 ± 1.4 (Martin et al. 1999)^*d*^	41 ± 1.7	Effects higher than those of Δ^9^-THC, enhancement of good mood, hunger, sleepiness, etc. (GasDank 2022).
			HPLC (SDPharmLabs 2022)	2411462-90-1 (acetoacetate)	-	-	Mellowing, relaxing, euphoriant, and energizing sensations with possible side effects including anxiety, dry mouth, dry or red eyes, increased appetite, increased heart rate, memory loss, slightly declined cognitive function, and slowed reaction times (Ward 2024)

^a^Absolute stereochemistry is not shown

^b^Where not specified, the CAS RN refers to the active stereoisomer (6aR,10aR)

^c^Stereochemistry is not specified

^d^Binding affinity is reported for the Δ^8^ version

### Search criteria for article selection

The literature search was performed mainly with three databases including SciFinder by “exact structure search”, Google Scholar, and Scopus to establish the scientific background. Technical notes from international companies, particularly focusing on analytical aspects, were also included. Search terms comprised chemical names and abbreviations of each cannabinoid, utilizing both old and new numbering systems for the benzopyran ring of THC. Keywords associated to the compounds of interest were “synthesis”, “analysis”, “CB1R affinity”, “in vitro activity”, “in vivo activity”, “pharmacology”, and similar. Additional literature was retrieved from the selected references. For the section on the recreational use, key websites included reddit.com, future4200.com, and bluelight.org, offering non-scientific information on each cannabinoid, including synthesis details, purchasing sources, and, most importantly, the psychoactive effects reported by the *psychonauts*, in particular the e-psychonauts. The term basically indicates people who voluntarily explore altered states of consciousness and share their experiences on dedicated websites (psychonaut websites).

A total of 151 scientific references were retrieved, while the remainder 72 come from web pages and non-scientific blogs.

### Scope of the review

The present review will offer a comprehensive overview of all THC-like cannabinoids, encompassing both non-natural and pseudo-natural variants. It will provide detailed insights into the manufacturing methods, analytical methodologies utilized for identification and quantification, the pharmacological aspects of each circulating molecule – restricted to in vitro CBR binding affinity and in vivo cannabimimetic activity – and, lastly, the effects experienced with the recreational use of such compounds.

As the scientific research cannot keep up with the recreational experimentation, some cannabinoids, either pseudo- or non-natural, have already been placed on the market and advertised as legal products claiming a natural origin. However, regardless of their origin, these products are advertised as new cannabinoids and often listed among the so-called “altnoids” meaning alternative cannabinoids different from Δ^9^-THC and CBD. All these compounds share the lack of scientific literature on the chemical synthesis and psychoactive effects. On the other hand, the psychonaut websites abound with information about the effects experienced by recreational users and recommendations on where to purchase these substances. For these compounds, a separate delineation of chemical, pharmacological, and analytical aspects is not feasible; thus, all available information from both grey and scientific literature will be consolidated in a single paragraph.

## Pseudo-natural cannabinoids (PNCs)

### Semi-synthetic pseudo-natural cannabinoids (SSPNCs)

Under this sub-group all Δ^9^-THC constitutional isomers can be found: the best-known naturally occurring isomer is Δ^8^-THC, in which the double bond is shifted to the adjacent position between C8 and C9. Other isomers are generated by the sequential shift of the double bond: Δ^6a,10a^-THC, Δ^6a,7^-THC, Δ^10^-THC, and Δ^9,11^-THC (also known as exo-THC). Some of these compounds are degradants of Δ^9^-THC under particular conditions of pH, but their natural existence in the cannabis plant, albeit in traces, cannot be ruled out. Several isomers of Δ^9^-THC can be identified in a typical chromatogram obtained by high performance liquid chromatography couple to high-resolution mass spectrometry (HPLC-HRMS), some of which are likely to correspond to the compounds mentioned above. Certain websites promoting or describing these compounds claim that they occur naturally in trace amounts in the plant (Schmidt 2022). Δ^7^-THC was excluded in this isomers list as its recreational use has not been documented, most likely because of either the lower binding affinity for CB1R and activity in vivo or the laborious synthesis. Although THCA is a fully natural cannabinoid, thus should not be seen as a pseudo-natural compound, it was included in this section due to its close affinity. Other SSPNCs included are HHC, along with its carboxylated precursor hexahydrocannabinolic acid (HHCA) and one of its hydroxyl metabolites (10-hydroxy-HHC), and dihydrocannabinol (DHC), a Δ^9^-THC with an additional double bond on C6a-C10a.

#### Tetrahydrocannabinolic acid (THCA) diamonds

(6a*R*,10a*R*)-1-Hydroxy-6,6,9-trimethyl-3-pentyl-6a,7,8,10a-tetrahydro-6*H*-benzo[*c*]chromene-2-carboxylic acid.

“Diamonds” is an umbrella word used to refer to the crystal form of a pure compound derived from *C. sativa*L. Available online for purchase are “Cannabis Diamonds”, “CBD Diamonds” and “THCA Diamonds” (MediaBros 2022). For the scope of this review only THCA Diamonds will be discussed.

##### Chemistry and synthesis

THCA diamonds (also “THC Diamonds” or “THCA/THC Crystals” and, possibly, “Cannabis Diamonds”) are basically made of almost pure (purity higher than 99%) Δ^9^-THCA.

*C. sativa* L. produces two different acidic forms of Δ^9^-THCA: Δ^9^-THCA-A showing the carboxylic group in *ortho* position to the aromatic hydroxyl function and Δ^9^-THCA-B with the carboxylic group in *para* position to the aromatic hydroxyl group. The former is the main enzymatic product of THCA synthase and is predominant in the plant, while the latter is only produced in small amount. Despite their structural similarities they show a substantial difference in the decarboxylation attitude (the B form is extremely more stable) and in the stability of the crystalline form (Filer 2021; Mechoulam et al. 1969).

In the absence of an extensive characterization of THCA diamonds available on the market, it is reasonable, from the authors’ point of view, to deduce that they should be mainly made of Δ^9^-THCA-A, being Δ^9^-THCA-B less available and less exploitable for recreational use.

Indeed, according to descriptions available online, THCA diamonds are obtained when the raw material undergoes cold extraction, mainly employing butane, repeated purification steps and lastly a slow removal of the solvent leading to crystallization of the isolated cannabinoid. When the process is correctly run it yields a pure final product with no hint of terpenes and flavonoids, thus some manufacturers add a mixture of them (the so called “diamond sauce”) to restore some entourage effect. This kind of product is also commercially known as “diamonds in sauce”) (Delphi 2022).

This production technique raises legal concerns. Due to reasons related to yield, the extraction is likely made from marijuana rather than hemp. This means the process would involve an illegal and untraceable product, leading to doubts about the safety guarantee of the final product.

Alternative synthetic paths may involve CBD as a starting reagent. At present, this is only discussed in specialized online forums, but it could become a feasible solution due to the absence of legal issues and the greater availability of CBD.

Apart from those risks posed by the high concentration of active substance, the main health issues arising from the consumption of diamonds are therefore due to the possible presence in the final product of solvents residues from the extraction and purification steps (Burb n.d.).

##### Pharmacology

Δ^9^-THCA is largely known for being the non-psychoactive cannabinoid originally produced by *C. sativa* L., then turned into its psychoactive counterpart through heat-induced decarboxylation. This is why recreational use of this plant has traditionally been based on smoking, today accompanied by other practices like dabbing, vaping, etc. Similarly, the preparation of Δ^9^-THC edibles always involves a cooking/baking step.

The non-psychoactive nature of Δ^9^-THCA has always been explained by the lack of binding affinity at the human CB1R, even if data in the scientific literature were not in accordance reporting an affinity sometimes equal to THC or 25-fold weaker or totally absent (McPartland et al. 2017). In their 2017 study on THCA’s affinity and efficacy at the human cannabinoid receptors *h* CB1R and *h*CB2R, McPartland et al. highlighted the importance of materials purity that had to be checked prior any test (McPartland et al. 2017). Indeed, THCA is particularly susceptible to losing the carboxylic group even when stored at freezer temperatures and they suggested that when *h*CB1R activity is found this is due to the presence of THC impurities rather than to THCA itself (McPartland et al. 2017).

A. Zagzoog et al. explored THCA’s biological activity in vitro and in vivo. In vitro tests evaluated its activity on each *h*CBR in Chinese hamster ovary (CHO) cell membranes through the displacement test using [^3^H]CP55,940 as reference material. Unlike Δ^9^-THC (*K*_*i*_ = 36 nm), the competition displacement assays indicated that THCA could not fully displace [^3^H]CP55,940 from *h*CB1R (*K*_*i*_ = 620 nM), but showed a small but significant displacement from *h*CB2R (*K*_*i*_= 1.3 nM), most likely indicative of a non-competitive binding (Zagzoog et al. 2020). In vivo evaluation was performed through the tetrad assays on any cataleptic, hypothermic, anti-nociceptive, locomotive, and anxiety-modifying activity. The study attributed to ∆^9^-THCA anti-nociceptive and hypolocomotive effects at 3 and 10 mg/kg and anxiolytic-like effects at 10 mg/kg (Zagzoog et al. 2020).

##### Identification and analysis

In recent years, several analytical techniques for the analysis of products derived from *C. sativa* L. have increasingly been tested as a consequence of the in-depth investigations on the pharmacological effects of the various compounds produced by the plant but also because its use is becoming progressively legal and accepted.

The two most common analytical methods are based on gas (GC) and liquid (LC) chromatography. In GC high temperatures in the injection port and in the oven turn Δ^9^-THCA into Δ^9^-THC, thus in the analytical result the specific contribution of each of them remains unknown. The only way to separately determine the two compounds is a derivatization step during sample preparation prior to GC analysis, which is not needed in the HPLC technique. In GC, chromatographic separation is often obtained through a low polarity stationary phase, as for instance made of 5% diphenyl- and 95% dimethyl polysiloxane, and specificity may be further increased with tandem GC (GC×GC). The chromatographic dimension may be coupled with a Flame Ionization Detector (FID) or a mass spectrometer (MS).

The rationale of the GC approach without derivatization, as mentioned above, is based on the conversion of Δ^9^-THCA into Δ^9^-THC. This represents a sensitive matter when the analysis is run for legal or forensic purposes. As this technique combines the undefined contributions of the carboxylated and decarboxylated compounds, the final analytical outcome loses its qualitative significance. Moreover, the presence of the illegal Δ^9^-THC in the analytical results does not necessarily imply that it was already present in the original sample, at least in the amount indicated by the numerical data, but rather derived from the conversion of the carboxylated precursor Δ^9^-THCA.

High performance liquid chromatography (HPLC) is the most suitable technique for the analysis of THCA. Being run at ambient temperature, it prevents its decarboxylation and allows for a separate identification and quantification of neutral Δ^9^-THC and acidic Δ^9^-THCA. The best separation between chromatographic peaks is obtained through a C_18_ stationary phase. The liquid chromatographer may be conventional or an ultra-high performance one with better separations at higher speed thanks to columns packed with stationary phases with particles smaller than 2 μm. It may be coupled to detectors, usually mass spectrometry (MS) or a UV/DAD (Diode Array Detector). The DAD is able to provide more details on the chromatographic peak improving method specificity as Δ^9^-THCA has an absorption spectrum that differs from that of its decarboxylated form. On the other hand, MS offers the greatest potential thanks to high-resolution detectors (HRMS such as QToF and Orbitrap) responsible of a higher specificity in case of extracts from complex matrices compared to UV/DAD. Moreover, HRMS enables the distinction between different compounds with the same nominal mass (isomers).

In light of the above, HPLC/UHPLC coupled to HRMS currently stands as the most powerful and versatile technique for the analysis of complex mixtures of cannabinoids. In particular, this approach allows for the determination of the exact mass of each compound, the independent quantification of both carboxylated and decarboxylated form of several cannabinoids (including THCA) without the need of derivatization. Consequently, it provides a more comprehensive characterization of each sample.

Several examples are present in the literature regarding the analysis of THCA in several matrices, including commercial products and biological specimens, employing both the GC-MS technique with derivatization (Nahar et al. 2020) and the HPLC-HRMS technique (Nahar et al. 2020). With specific focus on the analysis of THCA diamonds, it is not possible to find any scientific work, but some companies selling the product also provide the certificate of analysis. The most common routine technique employed in this case is HPLC coupled to either DAD or UV detector (SCLabs 2022; Analytical GP 2021; Inc. SAS 2023).

##### Recreational use

THCA diamonds are described as a very appealing substance intriguing the user both for its form and colour but above all because they are known as a concentrated legal form of THC (TheChronicBeaver 2021).

Online sites and personal blogs dealing with THCA diamonds explain their composition, production, the origin of the colour and warn about the possible presence of solvent residues. They advise how to use them as they need to undergo a decarboxylation process, thus eating them in the raw form does not produce an effect (Delphi 2022). Moreover, most people suggest that the best way to get the most of them is vaping or dabbing and recommend to consume them in progressively increasing doses (TheChronicBeaver 2021; Bortolazzo n.d.).

On the online forums, some people do not seem enthusiastic about THCA diamonds effects with respect to traditional cannabis products and suggest to blend them with terpenes or cannabis for a better experience confirming, from their perspective, the so called “entourage effect” (reddit.com. 2021). Others add more details and state that “white” diamonds produce a more “head” experience while diamonds “in sauce” produce a more “bodily” high and are more similar to the original cannabis plant material (Bluelight n.d.).

#### Δ^8^-Tetrahydrocannabinol (Δ^8^-THC)

(6a*R*,10a*R*)-6,6,9-Trimethyl-3-pentyl-6a,7,10,10a-tetrahydrobenzo[*c*]chromen-1-ol.

##### Chemistry and synthesis

Δ^8^-THC derives from an acid- or oxidatively promoted shift of the endocyclic double bond to the more thermodynamically stable position Δ^8^ (Razdan 1981). It is known since 1942 when it was synthesized by electrophilic cyclization of CBD and used in human studies (Adams 1942). Because of the Agricultural Improvement Act (Farm Bill Act), which include “all derivatives, extracts, cannabinoids, isomers, acids, salts and salts of isomers” in the definition of “hemp” (Agriculture Improvement Act of 2018 2018), Δ^8^-THC has been erroneously considered a legal product when it is derived from hemp CBD. The conversion reaction of CBD into Δ^9^-THC is known since the early 1960s thanks to Gaoni and Mechoulam’s studies (Gaoni and Mechoulam 1964) and a couple of years later they observed that either strong or mild acidic conditions in the reaction lead to different products (Gaoni and Mechoulam 1966). In particular, Δ^9^-THC is obtained from CBD upon treatment with HCl (0.0.5% in absolute ethanol) for 2 h, while Δ^8^-THC is obtained in relatively high yield by treating CBD with *p*-toluensulfonic acid (*p*TSA) for 18 h (Gaoni and Mechoulam 1966). Over the years the synthetic procedure for the manufacturing of Δ^8^-THC has not changed and the products circulating on the market are prepared according to the original procedure (Gaoni and Mechoulam 1966).

The major concern about the illicit manufacturing of Δ^8^-THC products is the presence of a number of contaminants, some of which are also toxic. Besides the presence of Δ^9^-THC in concentrations exceeding the legal limit, other contaminants can include Δ^7^-THC, Δ^10^-THC, Δ^11^-THC, 11-OH-CBD, 11-OH-THC, 5’-OH-CBD, 11,5’-diOH-CBD, 11,5’-diOH-THC, Δ^8^-iso-THC, Δ^4(8)^-iso-THC, and various substituted hexahydrocannabinols (HHCs), such as 9α-OH-HHC and 8-OH-iso-HHC (Kiselak et al. 2020), heavy metals in quantities non-compliant with the USP (copper, chromium and nickel), solvents (dichloromethane, methanol, ethyl acetate, and isopropanol) and several unknown cannabinoids (USCC 2021).

##### Pharmacology

Since its discovery, Δ^8^-THC was used in animal and human studies and found to exert a cannabimimetic activity similar to that of its isomer Δ^9^-THC (Adams 1942). The results of the in vitro activity are quite different among the experiments across the years, but it can be concluded that Δ^8^-THC has a slightly lower CB1R binding affinity compared to its regioisomer Δ^9^-THC (*K*_*i*_ = 41 ± 1.7 nM for Δ^9^-THC *vs. K*_*i*_ = 45 ± 12 nM for Δ^8^-THC (Martin et al. 1999)). Also, a different balance of CB1R vs. CB2R activation and a different formation rate of the active 11-OH metabolite contribute to a lower in vivo potency of Δ^8^-THC compared to Δ^9^-THC. However, further studies are required to clarify this difference, especially CB1R and CB2R binding affinity experiments in human and rodent under equivalent experimental conditions, which is important for assessing translatability of in vivo studies to humans (Tagen and Klumpers 2022).

##### Identification and analysis

Synthetic (-)-*trans*-Δ^8^-THC was characterized by proton and carbon nuclear magnetic resonance (^1^H NMR and ^13^C NMR) spectroscopy (Huang et al. 2010; Choi et al. 2004; Archer et al. 1977), hetero NMR (Bluelight n.d.) spectroscopy, infrared (IR) spectroscopy (Huang et al. 2010), UV-Vis (Hazekamp et al. 2005; De Backer et al. 2009) spectroscopy and mass spectrometry (MS) (Huang et al. 2010; Hazekamp et al. 2005).

In analytical investigations Δ^8^-THC behaves in a very similar way as Δ^9^-THC. Therefore, it is important to distinguish between Δ^9^-THC, Δ^8^-THC and all the other isomers. A great number of papers have been published on this topic and cover the analysis of Δ^9^-THC and Δ^8^-THC and in some cases other THC isomers in a wide range of matrices, including plant material, CBD oil, e-cigarette liquids, foods and beverages, but also biological specimens (blood, urine, oral fluid) (Tagen and Klumpers 2022).

For non-biological fluids, the most common analytical technique for the analysis of THC isomers, including Δ^8^-THC, is HPLC-UV (or HPLC-DAD) under reverse-phase conditions. Such methodology ensures good resolution between Δ^9^-THC and Δ^8^-THC (Citti et al. 2020; Micalizzi et al. 2021; Song et al. 2022). However, as THC isomers show very similar spectroscopic properties, such detection techniques lack specificity and are not able to separate very complex mixtures. HPLC-HRMS, besides the higher sensitivity compared to HPLC-UV, provides differentiation between the several isomers through the different mass fragmentation patterns and identification of synthetic side products (Kiselak et al. 2020; Pellati et al. 2018). Similarly, GC-MS is also able to distinguish between several THC isomers (Sams 2022).

In biological fluids Δ^8^-THC is generally detected and quantified as a chemical entity different from Δ^9^-THC by HPLC-MS/MS (Reber et al. 2022). For example, the method developed by Reber et al. also included the separation of exo-THC (Δ^9,11^-THC), ∆^6a,10a^-THC and ∆^10^-THC to exclude potential interfering compounds (Reber et al. 2022).

A comprehensive review on the chromatographic resolution of Δ^9^-THC isomers in different matrices has been recently published by La Maida et al. (2022).

##### Recreational use

According to informative websites, Δ^8^-THC produces a mild psychoactive experience that users describe as calming and euphoric. It can reduce feelings of nausea and provides many other effects like relaxation, euphoria and relief from pain. Most consumers have reported a noticeably milder experience compared to Δ^9^-THC together with sedative effects (Schmidt 2023). As a result, the side effects like paranoia are remarkably milder.

Nowadays, it is possible to find Δ^8^-THC in many cannabis and hemp-based products including distillate cartridges and syringes, vape cartridges, tinctures, oils, concentrates, gummies and edibles, beverages, and flowers (sprayed with Δ^8^-THC) (Schmidt 2023).

#### Δ^10^-Tetrahydrocannabinol (Δ^10^-THC)

(6a*R*,9*R*)-6,6,6a,9-Tetramethyl-3-pentyl-6a,7,8,9-tetrahydro-6*H*-benzo[*c*]chromen-1-ol *and* (6a*R*,9*S*)-6,6,6a,9-tetramethyl-3-pentyl-6a,7,8,9-tetrahydro-6*H*-benzo[c]chromen-1-ol.

##### Chemistry and synthesis

This isomer of Δ^9^-THC was originally synthesized by Srebnik et al. in 1984. As two epimers are possible, each one can be obtained with significant excess following two procedures starting from Δ^9^-THC: procedure A involves the use of *t*-pentyl potassium in toluene-hexamethylphosphoric triamide (HMPA) (6: 1) at reflux temperature, while procedure B requires the use of *n*-butyl-lithium in hexane (1.65 M) and HMPA at 0 °C (Srebnik et al. 1984). Basically, it is possible to start from CBD, which is converted into Δ^9^-THC and follow either one procedure or the other. Under the conditions of procedure B, an 8:1 mixture of (6a*R*,9*S*)-Δ^10^-THC and (6a*R*,9*R*)-Δ^10^-THC; on the other hand, procedure A leads to a 1:9 mixture of (6a*R*,9*S*)-Δ^10^-THC and (6a*R*,9*R*)-Δ^10^-THC respectively (Srebnik et al. 1984).

##### Pharmacology

Early studies on pigeons trained to discriminate between the presence and absence of Δ^9^-THC demonstrated that the (6a*R*,9*R*) epimer of Δ^10^-THC was the active compound in terms of cannabimimetic effects, while the (6a*R*,9*S*) epimer was inactive (Järbe et al. 1988). It is also reported that even the active epimer (9*R*) is less active that the active epimer of Δ^6a,10a^-THC (9*S*) (see next paragraph), which in turn is less active than Δ^9^-THC (Järbe et al. 1988). No data is available on the CB1R and CB2R affinity of the two epimers of Δ^10^-THC, thus no comparison with Δ^9^-THC can be made in terms of in vitro binding affinity.

##### Identification and analysis

Synthesis and full characterization of pure epimers (9*R*)-Δ^10^-THC and (9*S*)-Δ^10^-THC was performed by Srebnik et al. reporting optical rotations, UV, IR, NMR and MS spectra (Srebnik et al. 1984). The two epimers of Δ^10^-THC, along with those of Δ^6a,10a^-THC, were resolved at the baseline using an HPLC-UV method in normal phase (NP) conditions (95:5 hexane:isopropanol) (Williams et al. 2021). Moreover, the same isomers were successfully resolved using chiral stationary phases (CSPs) as recently reported by Umstead (2021). In details, the author used the CHIRALPAK IG-^3^ and the CHIRALPAK IB N-_3_CSP in NP conditions eluting either 95:5 hexane-ethanol or 90:10 hexane-isopropanol, though the two CSPs gave inverted elution orders (Umstead 2021).

Other methods include GC-MS applied to vaping liquids to distinguish between Δ^9^-THC, Δ^8^-THC, Δ^6a,10a^-THC, (9*R*)-Δ^10^-THC, (9*S*)-Δ^10^-THC, Δ^9,11^-THC, cannabinol (CBN), CBD, and olivetol (Ciolino et al. 2021), and lastly a bidimensional LC-MS/MS applied to e-cigarette cartridges, where the authors identified Δ^9^-THC, Δ^8^-THC, Δ^6a,10a^-THC, Δ^10^-THC, and CBD (Chan-Hosokawa et al. 2021).

##### Recreational use

Cannabinoids users describe Δ^10^-THC as an uplifting and energizing substance with a gentle head buzz (Schmidt 2023). Many users report that it enhances motivation, creativity, and cognitive function (Schmidt 2023). Reddit users claim similar effects to Δ^8^-THC, albeit less potent, that lead more to a “mind high” rather than a physical body high (reddit.com. 2020). Δ^10^-THC based products are increasingly spreading worldwide in the form of disposable pens, vape cartridges, gummies, tinctures and oils, dabbing syringes, chocolate bars, lollipops, shatter, flowers, and pre-rolls (sprayed with Delta-10 distillate) (Schmidt 2023).

#### Δ^6a,10a^-Tetrahydrocannabinol (Δ^6a,10a^-THC)

(*S*)-6,6,9-Trimethyl-3-pentyl-7,8,9,10-tetrahydro-6*H*-benzo[*c*]chromen-1-ol *and* (*R*)-6,6,9-trimethyl-3-pentyl-7,8,9,10-tetrahydro-6*H*-benzo[*c*]chromen-1-ol.

##### Chemistry and synthesis

This isomer was first synthesised by Adams and Baker in (1940). Condensation of ethyl 5-methylcyclohexanone-2-carboxylate with olivetol, followed by addition of methylmagnesium iodide, afforded the product as a colourless viscous oil in 78% yield (Adams and Baker 1940). In 2014 Rosati et al. published a one-pot heterogeneous synthesis of Δ^6a,10a^-THC (or Δ^3^-THC according to the old numbering) starting from an equimolar mixture of either (*R*)-(+)-pulegone or (*S*)-(−)-pulegone and olivetol in 1,2-dichloroethane (Rosati et al. 2014). The reaction was carried out in the microwave using either α-zyrconium sulphenylphosphonate [(α-Zr(O_3_PCH_3_)_1.2_(O_3_PC6H_4_SO_3_H)_0.8_], sulfuric acid supported on silica gel [H_2_SO_4_–SiO_2_], ytterbium triflate [Yb(OTf)_3_], or ytterbium triflate-ascorbic acid [YTACA 1:10] as catalyst (Rosati et al. 2014). Both epimers could be obtained with 49% and 47% yield for (9*R*)- and (9*S*)-Δ^6a,10a^-THC, respectively, and a purity of 98% for both compounds (Rosati et al. 2014).

##### Pharmacology

The dog ataxia test revealed that the two epimers and the racemic mixture behaves differently with the laevorotatory epimer being four to five times more active, and the dextrorotatory epimer two to three times less active than the racemate, although no comparison was made with Δ^9^-THC (Adams et al. 1942). Later, Hollister et al. tested the epimers and the racemate in humans, observing that the (9*S*) epimer was the active one producing one third to one sixth the effects of Δ^9^-THC (Hollister et al. 1987). On the other hand, the (9*R*) epimer did not bring any psychoactive effects as the activity did not change when the two epimers were administered together in a 1:1 mixture (Hollister et al. 1987). In 2014, Rosati et al. measured the CB1R and CB2R binding affinity of both epimers, revealing that CB1R affinity of (9*S*)-Δ^6a,10a^-THC was higher (*K*_*i*_ = 5 nm) than that of Δ^9^-THC (*K*_*i*_ = 22 nm), while the one of the (9*R*) epimer was in the range of that of Δ^9^-THC (*K*_*i*_ = 29 nm). Both epimers showed similar affinity for CB2R (*K*_*i*_ = 17 and 18 nm for (9*S*)- and (9*R*) epimer, respectively), which was higher than that of Δ^9^-THC (*K*_*i*_= 46 nm) (Rosati et al. 2014).

##### Identification and analysis

Identification of pure enantiomers (*R*)-(+)-Δ^6a,10a^-THC and (*S*)-(−)-Δ^6a,10a^-THC was described by Srebnik et al. reporting optical rotations, UV, IR, NMR and MS spectra (Srebnik et al. 1984).

The few analytical methods for the analysis of Δ^6a,10a^-THC reported in the literature match those described above for Δ^10^-THC (Williams et al. 2021; Umstead 2021; Ciolino et al. 2021; Chan-Hosokawa et al. 2021).

##### Recreational use

On informative websites, it is reported that Δ^6a,10a^-THC has the same effects as Δ^10^-THC, thus milder than Δ^9^-THC (Schmidt 2022). Some people report uplifting and energizing effects (similar to Δ^10^-THC); many users also report a heightened sense of mental clarity and focus without paranoia and anxiety (Schmidt 2022).

As this compound is quite new on the recreational market, it is sometimes mislabelled as Δ^10^-THC (and vice versa).

#### 10-Oxo-Δ^6a,10a^-Tetrahydrocannabinol (10-Oxo-Δ^6a,10a^-THC)

(*R*)-1-Hydroxy-6,6,9-trimethyl-3-pentyl-8,9-dihydro-6*H*-benzo[*c*]chromen-10(7*H*)-one *and* (*S*)-1-hydroxy-6,6,9-trimethyl-3-pentyl-8,9-dihydro-6*H*-benzo[*c*]chromen-10(7*H*)-one.

##### Chemistry and synthesis

In 1975, Friedrich-Fiechtl and Spiteller isolated 10-oxo-Δ^6a,10a^-THC from a hashish extract by preparative thin layer chromatography (TLC) (Friedrich-Fiechtl and Spiteller 1975). Two years later, Kettenes-van den Bosch and Salemink discovered this compound in cigarette smoke condensate, specifically in the neutral fraction (Kettenes-van den Bosch and Salemink 1977). Lewis et al. identified the compound in decarboxylated medicinal cannabis extracts and highlighted its absence in the native extracts, suggesting that the product is released upon heating (Lewis et al. 2017). No record is present in the literature regarding the synthesis of 10-oxo-Δ^6a,10a^-THC, but it rather seems it can be isolated from cannabis extracts following decarboxylation. It is likely a decomposition product of Δ^9^-THC, although information on the enantiomeric composition of the spontaneously formed product is currently unavailable.

##### Pharmacology

No research has been conducted on the pharmacology of this cannabinoid but it is believed to act as Δ^9^-THC on CB1R.

##### Identification and analysis

The pure compound has been characterized by GC-MS, NMR, FT-IR (Friedrich-Fiechtl and Spiteller 1975; Kettenes-van den Bosch and Salemink 1977), and HPLC-MS in RP conditions (Lewis et al. 2017). Its quantification has been achieved by GC-FID in cannabis smoke condensate after separation of three fractions: acidic, phenolic and neutral, the latter being the one containing this specific compound (Friedrich-Fiechtl and Spiteller 1975; Kettenes-van den Bosch and Salemink 1977).

##### Recreational use

10-Oxo-Δ^6a,10a^-THC is not widespread for recreational use, but some websites promote the compound supposing it has similar properties as Δ^9^-THC (Hewett 2022; What is 10-Oxo-Δ6a(10a)-tetrahydrocannabinol (OTHC)? 2023).

#### Δ^9,11^-Tetrahydrocannabinol (Δ^9,11^-THC)

(6a*R*,10a*R*)-6a,7,8,9,10,10a-Hexahydro-6,6-dimethyl-9-methylene-3-pentyl-6*H*-dibenzo[*b*,*d*]pyran-1-ol.

##### Chemistry and synthesis

Δ^9,11^-THC is known since the late 1960s, when it was first synthesized by Fahrenholtz et al. from olivetol and diethyl α-acetoglutarate leading to the racemic mixture of the compound (Fahrenholtz et al. 1967). This type of synthesis would make it a completely synthetic Δ^9^-THC derivative, but another synthetic procedure was developed by Wildes et al. from either Δ^8^-THC or Δ^9^-THC with potassium tricyclopentylcarbinolate to obtain the pure stereoisomeric forms in 40–45% yield (Pitt et al. 1971). Further improvements were made in the late 1980s using Δ^8^-THC as the precursor for the synthesis, which is converted into a 3:1 separable mixture of (9*R*)-chlorohexahydrocannabinol ((9*R*)-Cl-HHC) and (9*S*)-Cl-HHC by adding gaseous hydrochloric acid and zinc chloride (Banijamali and Makriyannis 1988). The product was obtained in 65% overall yield by HCl elimination with potassium *t*-amilate (Banijamali and Makriyannis 1988). Either Δ^8^-THC or Δ^9^-THC can be used as precursors as they both are converted into a mixture of (9*R*)-Cl-HHC and (9*S*)-Cl-HHC with excess of the former (Banijamali et al. 1998).

There is no record on the natural occurrence of such cannabinoid, although it is reported also as a very small impurity of Δ^9^-THC isomerization after decarboxylation of the plant material (Cid and Van Houten 2015). Nonetheless, it would still represent a minor component whose extraction would not be cost-effective. In light of the above, the synthesis of Δ^9,11^-THC, specifically the semi-synthesis, is the most feasible way starting from the legal CBD.

##### Pharmacology

Δ^9,11^-THC is not as psychoactive as Δ^9^-THC. Indeed, its cannabimimetic activity was found to be one twentieth of the parent compound when tested on rats for their activity in the cage (Christensen et al. 1971) and one fifteenth in a multiple test (locomotor activity, tail-flick latency, hypothermia, ring immobility) on rats (Compton et al. 1991). These results are in line with its CBR (supposedly CB1R) binding affinity (*K*_*i*_ [Δ^9,11^-THC] = 334 nm, *vs. K*_*i*_ [Δ^9^-THC] = 218 nM) (Compton et al. 1991). However, it is interesting to note that Δ^9,11^-THC is only 4-fold less potent than Δ^9^-THC in the generation of the antinociceptive response in the mouse (Compton et al. 1991).

In another study, Δ^9,11^-THC resulted 100-fold less potent than Δ^9^-THC in producing hypothermia, analgesia, lethality and in reducing spontaneous activity in mice, while it did not show any cannabimimetic effects in dogs (static ataxia, hyperreflexia, prancing and tail-tuck) and rhesus monkeys (ptosis, sedation and ataxia) (Beardsley et al. 1987).

Besides the lower affinity for CB1R, the lower potency of Δ^9,11^-THC compared to Δ^9^-THC can be also justified by the failure to produce the well-known metabolite 11-hydroxy-THC, which has even stronger effects than the parent compound Δ^9^-THC.

##### Identification and analysis

Besides the full characterization of the pure compound by NMR, FT-IR, and MS (Fahrenholtz et al. 1967), Δ^9,11^-THC was analyzed in mixture with Δ^9^-THC and Δ^8^-THC by HPLC-UV in NP conditions by Banijamali and Makriyannis (1987), in mixture with other cannabinoids by HPLC-UV in RP conditions (Krepich et al. 2020; Franklin and Wilcox 2019), and in vaping liquids by HPLC-DAD in RP conditions (Ciolino et al. 2021).

##### Recreational use

Δ^9,11^-THC can be found in several products like vaping liquids, cartridges, sauces and waxes with various names including delta-11-THC and exo-THC in combination with other cannabinoids (Ciolino et al. 2021; reddit.com 2021). However, the name delta-11-THC is also used improperly to indicate 11-hydroxy-THC, which is the primary Δ^9^-THC metabolite. Exo-THC is advertised as an exotic component in hemp derived products (reddit.com 2021).

There are not many reports on the recreational experience of exo-THC, highlighting the quite recent attention to this cannabinoid, but a user on reddit.com declared that consuming a “full spectrum” vape cartridge labelled with CBD, cannabichromene (CBC), cannabigerol (CBG), CBN and other minor cannabinoids including exo-THC (8.27%), cannabitriol (CBT) (10.5%), and cannabielsoin (CBE) (5.72%) led to a “good spacey feeling” and slow reaction time, though keeping a clear head sensation (reddit.com. 2023). However, given the complexity of the cannabinoid mixture in the product, the effects might be ascribed to some other compound.

#### 11-Hydroxy-tetrahydrocannabinol (11-OH-THC)

(6a*R*,10a*R*)-9-(Hydroxymethyl)-6,6-dimethyl-3-pentyl-6a,7,8,10a-tetrahydro-6*H*-benzo[*c*]chromen-1-ol.

##### Chemistry and synthesis

11-Hydroxy-Δ^9^-THC (11-OH-THC) is the primary metabolite of Δ^9^-THC formed in the liver by the enzymatic activity of the CYP450 family (in particular CYP2C9 and CYP3A4) (Huestis 2005). Both oral consumption and inhalation of either the parent compound Δ^9^-THC or THC-containing products lead to the formation of this metabolite.

The chemical synthesis of the pure compound is neither facile nor high-yielding (Pitt et al. 1975), even starting from the legal CBD (Kiselak et al. 2020). Therefore, the only efficient way of making 11-OH-THC is using either rat microsomes (Wall et al. 1970) or liver homogenates (Wall 1971;  Nilsson et al. 1970), which lead to a 30% yield for the product.

##### Pharmacology

11-OH-THC is known to exert a several fold higher potency compared to the parent compound Δ^9^-THC both in vitro (with a *K*_*i*_ of 0.37 nm vs. 35 nm of Δ^9^-THC) (Zagzoog et al. 2022) and in vivo (Lemberger et al 1972, 1973). In a double-blind randomized experiment on humans, the 11-OH metabolite was reported to produce a greater psychoactive high in all subjects compared to Δ^9^-THC (Lemberger et al. 1973). In the same work, the intravenous administration of 11-OH-THC led to pronounced psychologic and pharmacologic effects within 2–3 min (marked tachycardia, intense psychologic high, etc.) (Lemberger et al. 1973). On the other hand, the intravenous administration of Δ^9^-THC had qualitatively similar effects, though not reaching their peak until 15–30 min (Lemberger et al. 1973). This and other studies, such as that of Lemberger et al. (1972), strongly support the hypothesis that the psychoactivity of Δ^9^-THC comes from its active metabolite since the effects of the latter show a good temporal correlation with the plasma levels of its metabolite.

##### Identification and analysis

Numerous validated methods are reported in the literature for the determination of 11-OH-THC in several matrices, usually in combination with Δ^9^-THC and other metabolites like Δ^9^-THC carboxylic acid (11-THC-COOH) and sometimes the glucuronic derivative by either GC-MS (Andrenyak et al. 2017; , Purschke et al. 2016) or LC-MS/MS (Reinstadler et al. 2023; Simões et al. 2011). Notwithstanding their very high sensitivity, all methods have been developed for the determination of THC metabolites in biological specimens, such as urine (Morisue Sartore et al. 2022), blood (Lo Faro et al. 2022), serum (Pichini et al. 2021), plasma (Manca et al. 2022), hair (Lo Faro et al. 2022) and oral fluid (Gorziza et al. 2023), not for the detection of such compounds in commercial products. Nonetheless, KCA Laboratories have tested a product claiming to contain 11-OH-Δ^8^-THC for purity, though not specifying the methodology employed, and the manufacturer published the certificate of analysis (KCALabs 2023). However, no other analytical method has been published for the application to matrices different from the biological specimens.

##### Recreational use

Until a few months ago, 11-OH-THC based products seemed not to be actually sold on the market, but only advertised to attract the attention of consumers with “exotic” and “alternative” cannabinoids. Indeed, all products advertising the alleged cannabinoid 11-OH-THC have actually turned out to be fakes after chemical analysis. In October 2023, the first product containing over 96% 11-OH-Δ^8^-THC as distillate manufactured by 3CHI reached the recreational market (KCALabs 2023; 3CHI 1959). Although most consumers are aware the products they buy claiming high concentration of 11-OH-THC do not actually contain this cannabinoid, some of them reported quick onset of the effects and a strength somehow close to THC, along with the negative effects like an accelerated heartbeat and racing thoughts (reddit.com. 2023). However, due to the presence of other cannabinoids in the products it is difficult to ascribe such effects solely to the alleged 11-OH-THC.

#### Hexahydrocannabinol (HHC)

(6a*R*,9*R*,10a*R*)-6,6,9-trimethyl-3-pentyl-6a,7,8,9,10,10a-hexahydrobenzo[*c*]chromen-1-ol and (6a*R*,9*S*,10a*R*)-6,6,9-trimethyl-3-pentyl-6a,7,8,9,10,10a-hexahydrobenzo[*c*]chromen-1-ol.

Research groups have always been interested in the potentialities of hexahydrocannabinol (HHC) and its saturated analogues but today it has gained an important role also in the market for recreational substances (EMCDDA technical expert meeting on hexahydrocannabinol (HHC) and related cannabinoids [press release] 2022). Indeed, the oversupply of CBD enriched extracts and its use as a chemical precursor for several SSCs have led manufacturers to offer to the market a new series of cannabinoids exploiting synthetic routes known in the scientific literature for decades. Among this series of cannabinoids, HHC may be seen as an exotic compound in the cannabis consumer market but it is not exactly a new cannabinoid as it was discovered in 1940 by Adams and Todd in their laboratories while exploring with the hydrogenation reaction on the THC molecule in marijuana (Adams et al. 1940). Therefore, during the last two years, HHC has been openly sold on the internet websites as a “legal” and cheaper alternative to THC and cannabis (Graziano et al. 2023).

##### Chemistry and synthesis

HHC has three stereogenic carbon atoms, and all eight stereoisomers have been discussed in the chemical literature. Of these, only the semi-synthetic (6a*R*,9*S*,10a*R*)-HHC (9α-HHC) and (6a*R*,9*R*,10a*R*)-HHC (9β-HHC) epimers have attracted attention, and only these isomers appear to have been encountered in marketed products.

The first total stereoselective synthesis of natural (9*R*)-HHC and its unnatural (9*S*)-HHC diastereomer was developed by Tietze starting with 5-pentylcyclohexane-1,3-dione and optically pure citronellal via an intramolecular Diels-Alder reaction and aldol condensation followed by aromatization and elimination along a two-step reaction (Tietze et al. 1982). However, already in 1940 Adams performed the semi-synthesis of HHC by hydrogenation of Δ^6a,7^-THC (Adams et al. 1940).

The semi-synthesis has been revised with several catalysts obtaining different yields and epimeric ratios (Gaoni and Mechoulam 1966; Cornia et al. 1989; Collins et al. 2023; Nasrallah and Garg 2023). In particular, the Garg’s group employed tri(acetylacetonato)iron(III) as the hydrogen atom donor catalyst for the radical reduction reactions in combination with thiophenol and sylilbenzene to reduce Δ^8^-THC (Nasrallah and Garg 2023). Under these conditions, the mixture of diastereomers afforded 77% of yield and an epimeric ratio of 11:1 ((9*R*)-HHC:(9*S*)-HHC).

##### Pharmacology

Since its discovery, HHC has been extensively studied both in vitro and in vivo for its pharmacologic and/or psychoactive effects although part of these studies, especially the oldest ones, are affected by the uncertain composition of the sample, thus their results are of difficult interpretation.

In vitro biological studies of HHC (both racemic mixtures and single epimers) dealt with its interaction with several different receptors and biochemical mechanisms such as cannabinoid receptors, opioid receptors, RPA1 ion channel activation, acetylcholinesterase inhibition, cytotoxicity to human lung fibroblasts and anticancer properties in four pancreatic cancer cells. Interestingly the 9β epimer generally shows a higher activity with respect to its 9α counterpart (Ujváry 2023).

In vivo tests (rabbit corneal reflex, tetrad test on mice, rhesus monkey behaviour, etc.) have been performed to assess cannabimimetic activity in comparison to Δ^9^-THC. Tests on rhesus monkeys, in particular, demonstrated that the cannabimimetic activity resided mainly, if not solely, in the laevorotatory 9β-HHC, with effects like ataxia, stupor, full ptosis, immobility, long lasting crouched posture, and absence of reaction (Edery et al. 1972; Mechoulam et al. 1980). Recent preliminary studies based on mouse tetrad assay confirmed the predominant cannabimimetic activity of the 9β epimer of HHC (Russo et al. 2023).

An important issue that HHC shares with all other commercialised semi-synthetic products is the possible contamination due to the synthetic pathway. Indeed, traces of heavy metals from hydrogenation along with byproducts of the other synthetic steps can be found in the final product because of the variable manufacturer’s expertise, differences in purification procedures and the absence of quality control (Ujváry 2023). Even the presence of an “analytical certificate” should not be considered a guarantee of product quality; indeed, the phenomenon of mislabelled products is currently widespread. For example, a GC-MS analysis revealed that a product sold as “HHC” in the United States was actually a mixture of Δ^8^-THC, Δ^9^-THC, Δ^6a,10a^-THC, and CBN (Sams 2022).

##### Identification and analysis

Being one of the first investigated phytocannabinoid derivatives, HHC’s behaviour in the application of the most common analytical techniques seems, at present, well understood.

A precise separation of the two 9α/9β HHC epimers may be easily obtained by chromatography (TLC, GC and HPLC). Since the early studies, the relationship between the chemical structure and GC retention time of several common cannabis constituents and synthetic cannabinoids, (including HHC, hexahydrocannabivarinol (HHCV), and other linear and branched HHC homologs) were investigated with either unmodified or trimethylsilyl (TMS)-derivatized analyte (Vree et al. 1972, 1973). HPLC demonstrated the same versatility as GC in separating both phyto- and synthetic cannabinoids and the two HHC epimers as well (Stothard et al. 2023).

The detector of choice for structure identification is mass spectrometry. Fragmentation patterns in the mass spectra of the 9α and 9β epimers of HHC are virtually identical; hence, in the analysis of real samples the stereochemical composition may be achieved only by GC/HPLC-MS analysis followed by a confirmation run of a standard of known epimeric purity. In this regard, a very recent work by Kobidze et al. describes the development of the first LC-MS/MS stereoselective bioanalytical method using a CSP to quantitatively detect (9*R*)-HHC and (9*S*)-HHC and their metabolites in human blood, oral fluid and urine (Kobidze et al. 2024).

Another long-tested technique is UV-visible spectroscopy which gives spectrum of HHC with main absorption bands at λ_max_272 and 282 nm in the UV region (Ujváry 2023).

^1^H NMR spectroscopy is also useful to discriminate between the two epimers (Ujváry 2023). Solvent effects have been associated to the chemical shift of the C10 and C10a protons adjacent to the phenolic moiety.^13^C NMR spectra distinguish between the 9α and 9β stereoisomers since the carbon atoms of the cycloalkane fragment appear at different chemical shifts for the two epimers. Except for C6a, the chemical shift of all carbon atoms of the cycloalkane moiety of the 9β epimer is shifted downfield.

Finally, several different radioimmunoassay (RIA) methods have been developed over the years and primarily for the detection of Δ^9^-THC, but they all show a certain degree of cross-reactivity with other cannabinoids and their metabolites, including HHC, even if the latter is of unstated epimeric purity (Ujváry 2023). For this reason, it seems unlikely that an HHC specific immunoassay technique may be available in the future.

##### Recreational use

During the last two years, HHC has been freely sold by internet websites as a “legal” replacement to THC and cannabis in a range of highly attractive branded and unbranded products, some of which are sold as “legal highs”.

Reported opinions describe HHC effects on average comparable to those of Δ^9^-THC (sedation and relaxation, euphoric high and a smooth, calming experience, less energetic and more cerebral) and only sporadic episodes of side effects like psychosis, uncontrolled tremors, and so forth (reddit.com 2023; Schmidt 2023; reddit.com 2023). The intensity of the effects can vary from person to person. Apparently, the majority of users vape it through e-cigarettes or smoke it in herbal matrices but gummies are also reported (reddit.com. 2023; reddit.com. 2023).

#### 10-Hydroxy-hexahydrocannabinol (10-OH-HHC)

(6a*R*,9*R*,10*S*,10a*R*)-6,6,9-Trimethyl-3-pentyl-6a,7,8,9,10,10a-hexahydro-6*H*-benzo[*c*]chromene-1,10-diol *and* (6a*R*,9*S*,10*R*,10a*R*)-6,6,9-trimethyl-3-pentyl-6a,7,8,9,10,10a-hexahydro-6*H*-benzo[*c*]chromene-1,10-diol.

##### Chemistry and synthesis

It exactly refers to (10*S*)-hydroxy-(9*R*)-hexahydrocannabinol, also known as 10α-hydroxy-(9*R*)-hexahydrocannabinol), a cannabinoid actually isolated from cannabis (Ahmed et al. 2015). It is also a known minor metabolite of HHC (Harvey and Brown 1991)and its structure is known since 1980 when it was first synthesized by Mechoulam (Mechoulam et al. 1980). Companies selling this cannabinoid claim to follow a semi-synthetic route starting from CBD, but they do not give any other information (Future4200 2023).

##### Pharmacology

Early studies on rhesus monkeys showed that the (10*S*,9*R*)- isomer is able to produce psychoactive effects already at 0.5 mg/kg (Mechoulam et al. 1980). No studies are present on its in vitro CB1R binding affinity.

##### Identification and analysis

Identification and differentiation of isomers can be achieved by GC-MS (both low and high-resolution), LC-MS, and NMR (Cayman 2023). In particular, although MS spectra are very similar, chromatographic retention times are slightly different with both techniques (GC and LC). In LC, with a linear gradient of methanol, the (10*R*,9*S*) isomer eluted first. NMR, particularly HMBC, COSY and NOESY, proves to be essential to distinguish axial and equatorial position of atoms.

##### Recreational use

In the Reddit forums it is stated that the effects are more or less the same as HHC itself, although a bit shorter in duration. Additionally, it is easier to handle as it is a crystalline solid (reddit.com. 2023).

#### Hexahydrocannabinolic acid (HHCA)

(6a*R*,9*R*,10a*R*)-1-Hydroxy-6,6,9-trimethyl-3-pentyl-6a,7,8,9,10,10a-hexahydro-6*H*-benzo[*c*]chromene-2-carboxylic acid *and* (6a*R*,9*S*,10a*R*)-1-hydroxy-6,6,9-trimethyl-3-pentyl-6a,7,8,9,10,10a-hexahydro-6*H*-benzo[*c*]chromene-2-carboxylic acid.

Hexahydrocannabinolic acid (HHCA) is the carboxylic acid analogue of HHC, analogous to THCA in relation to THC. Limited information is available about this cannabinoid, except for its synthesis and practical application. A semi-synthetic process for HHCA has been patented, starting with a crude cannabis essential oil that undergoes hydrogenation (HHCA is indicated as HTHCA) (Scialdone 2016). However, the epimeric composition of the final product is not mentioned.

No literature records are available on the pharmacological activities and analytical methods for the identification and quantification of the carboxylated form. However, it is not intended for use in its current state, as HHCA will undergo decarboxylation upon heating to be converted into HHC, which is the form actually consumed. It appears that the carboxylated species is much more stable than its decarboxylated counterpart, as it exists in a crystalline form, making it easier to handle and avoiding the stickiness associated with a distillate (reddit.com. 2023).

#### 7,8-Dihydrocannabinol (DHC)

6,6,9-Trimethyl-3-pentyl-7,8-dihydrobenzo[*c*]chromen-1-ol.

DHC is less common on forums and indicates 7,8-dihydrocannabinol with a structure similar to that of Δ^9^-THC but with an additional double bond on the C6a-C10a position (Jagannathan 2020). The semi-synthetic process was described in a patent starting from Δ^9^-THC, which could be easily obtained from CBD (as seen in the above paragraphs), and treating with 3,5-di-tert-butyl-1,2-benzoquinone (Loewinger et al. 2022). The reaction carried out at very low temperature (-40 °C) can afford pure DHC avoiding the formation of CBN, which is thermodynamically more stable (Loewinger et al. 2022).

Although this is expected to have the same pharmacological effects of Δ^9^-THC, it results more reactive to undergo liver metabolism (Jagannathan 2020) and be converted to 11-OH-THC, which is notedly more potent than the parent compound.

### Synthetic pseudo-natural cannabinoids

Under this sub-group all Δ^9^-THC homologues can be found, starting from those with different length of the alkyl side chain to their hydrogenated derivatives. All the compounds included in this section are of recent discovery, including the Δ^9^-THC analogues identified by the authors’ group (Linciano et al. 2020a; Linciano 2020b; Citti et al. 2019).

SPNCs, although being naturally occurring, cannot be obtained by a semi-synthetic process starting from CBD or Δ^9^-THC as the elongation of the alkyl side chain is not straightforward starting from these precursors. Therefore, a fully synthetic protocol is required to afford the desired products, generally starting from the resorcinol with the appropriate side chain length. Once the THC analogue is obtained, it can be easily hydrogenated to obtain the corresponding HHC derivative.

All Δ^9^-THC homologues sold for recreational use are generally not tested for purity, thus they can come in either pure Δ^9^ or Δ^8^ form or as a mixture of the two.

For all HHC analogues no scientific literature could be retrieved, only information on their recreational use was rather available.

#### Δ^9^-Tetrahydrocannabutol (Δ^9^-THCB)

(6a*R*,10a*R*)-3-Butyl-6,6,9-trimethyl-6a,7,8,10a-tetrahydro-6*H*-benzo[*c*]chromen-1-ol.

##### Chemistry and synthesis

Until a few years ago Δ^9^-THCB had only been detected and characterized by means of mass spectrometry in the plant matrix by Harvey in (1976). Then, in 2020 Linciano et al. identified Δ^9^-THCB in samples of *C. sativa *L., Italian FM2 medicinal variety, and delivered a full characterization through UHPLC-HRMS (Orbitrap), NMR, UV, and circular dichroism spectra (Linciano et al. 2020a).

In order to confirm the exact structure with absolute stereochemistry, the authors performed a stereoselective synthesis starting from a Friedel-Craft allylation of 5-butylbenzene-1,3-diol with (1*S*,4*R*)-1-methyl-4-(prop-1-en-2-yl)cycloex-2-enol (*p*TSA as catalyst). The reaction led to (−)-*trans*-CBDB, which was turned first into Δ^9^-THCB (not isolable at this step) and then, being more stable, quantitatively into (−)-*trans*-Δ^8^-THCB. By successive addition and removal of hydrochloric acid (respectively with ZnCl_2_ as catalyst and potassium *tert*-amylate as base), (−)-*trans*-Δ^8^-THCB was quantitatively converted into (−)-*trans*-Δ^9^-THCB (91% yield) (Linciano et al. 2020a).

##### Pharmacology

The only literature records on the pharmacological behaviour of Δ^9^-THCB are available from the work by Linciano et al. that reports both in vitro and in vivo investigation of the cannabimimetic properties of this cannabinoid (Linciano et al. 2020a).

The *h*CB1R and *h*CB2R binding affinity was tested in a radioligand test based on the displacement of radiolabeled [^3^H]CP55940 from *h*CB1R or [^3^H]WIN 55212-2 from *h*CB2R (Linciano et al. 2020a). Compared to Δ^9^-THC, Δ^9^-THCB showed a three-fold higher affinity for *h*CB1R (*K*_*i*_ = 15 nM for Δ^9^-THCB *vs. K*_*i*_ = 41 nM for Δ^9^-THC) and similar affinity for *h*CB2R (Linciano et al. 2020a).

In vivo cannabimimetic effects were evaluated through the tetrad tests in mice, which showed only a partial interaction of Δ^9^-THCB with the CB1R in the same fashion as Δ^9^-THC (Linciano et al. 2020a).

##### Identification and analysis

In order to characterize Δ^9^-THCB, Linciano et al. applied the main traditional techniques (HPLC-HRMS, ^1^H and ^13^C NMR, UV spectroscopy) and the more advanced approach based on metabolomics, an innovative tool particularly useful in simultaneously identifying the impressive number of phytocompounds present in *C. sativa*L (Linciano et al. 2020a).

At the time of writing, unlike what has been done for other cannabinoids, no specifically validated analytical methods for Δ^9^-THCB are described in the literature. It is therefore reasonable to assume that the same approaches are applicable to the analysis of Δ^9^-THCB in commercial products for recreational use, herbal products and biological samples. Indeed, a certificate of analysis of a cannabis food product (chocolate bar) reports a series of cannabinoids including Δ^9^-THCB analysed using a certified HPLC method (detector not specified) (SDPharmLabs 2023).

##### Recreational use

Δ^9^-THCB is commercially available online as an isolate (declared purity 96,9%) for the business to business market (Pharmabinoid 2023) and as in all other forms already encountered for other cannabinoids (tinctures, gummies and vape liquids) for retail (TheCalmLeaf 2023; Binoid n.d.). To the authors’ knowledge, no investigation has been done on its exact composition (i.e. whether it is pure Δ^9^-THCB or Δ^8^-THCB or a mixture thereof).

By type and duration of effects, users declare that its potency is either comparable to or higher than that of Δ^9^-THC (Brandrup 2023; Future4200 2022). They also refer of quick sensations of euphoria, mental energy and uplift as well as feelings of bodily relaxation and calmness (Brandrup 2023). Moreover, especially long standing users complain about the short duration of the effects and that they are not particularly distinctive in case of developed tolerance (reddit.com. 2023).

#### Δ^9^-Tetrahydrocannabihexol (Δ^9^-THCH)

(6a*R*,10a*R*)-3-Hexyl-6,6,9-trimethyl-6a,7,8,10a-tetrahydro-6*H*-benzo[*c*]chromen-1-ol.

##### Chemistry and synthesis

Among the homologues of Δ^9^-THC with varying lengths of the alkyl side chain, (−)-*trans*-Δ^9^-tetrahydrocannabihexol ((−)-*trans*-Δ^9^-THCH) emerged as the latest addition to the series, completing the spectrum after the discovery of butyl- and heptyl-side chain compounds. Traditionally, it was believed that the biosynthesis of cannabinoids in *C. sativa*L. could only lead to compounds with an odd number of carbon atoms in the side chain, with even numbers arising from subsequent fungal ω-oxidation (Hanuš et al. 2016). However, recent advances in metabolomics analysis in an untargeted fashion using UHPLC-HRMS and, in particular, UHPLC interfaced to an Orbitrap mass spectrometer with heated electrospray ionization source (UHPLC-HESI-Orbitrap) have enabled a thorough investigation of all compounds, even in trace amounts, in cannabis varieties. In 2020, this technology led to the identification of Δ^9^-THCH in the Italian medicinal variety FM2 (Linciano et al. 2020a).

The identity of (−)-*trans*-Δ^9^-THCH in FM2 extracts was confirmed through stereoselective *in house* synthesis, demonstrating a complete overlap of analytical data obtained from UHPLC-HESI-Orbitrap and ^1^H and ^13^C NMR spectroscopy. The synthetic pathway involved the condensation of 5-hexyl-resorcinol with (1*S*,4*R*)-1-methyl-4-(prop-1-en-2-yl)cycloex-2-enol, catalyzed by *p*TSA. The initial product is the Δ^8^-THC analogue generated from a CBD intermediate and subsequently converted into its Δ^9^ chlorinated derivative. The latter undergoes selective elimination of the chlorine atom from C9 on the terpene moiety to yield the desired Δ^9^-THC product. Due to the time-consuming nature and low yields of this procedure, the authors stopped the process at the stage where CBDH is still present, preventing the conversion of Δ^9^-THCH into Δ^8^-THCH. Subsequent separation of the two single homologues was achieved through semi-preparative LC (Linciano et al. 2020a).

In the hypothesis that the same production procedure is used to obtain recreational Δ^9^-THCH, it is reasonable that what is labelled as THCH could be a mixture of Δ^8^ and Δ^9^-THCH in varying proportions.

##### Pharmacology

To the best of the authors’ knowledge, there is no research article in the scientific literature specifically addressing the in vivo or in vitro evaluation of the psychoactive effects of Δ^9^-THCH. However, drawing on information available for related compounds and consumer reports, it is reasonable to consider its interaction with *h*CB1R as highly likely, given its increased lipophilicity. A comprehensive comparative study, encompassing in vivo and in vitro approaches, of the entire *n*-alkyl side chain THC series could provide valuable insights into the role of the side chain in receptor interaction.

Brown and Harvey conducted an evaluation of the metabolism of Δ^8^ and Δ^9^-THCH in mice, revealing that their pharmacokinetics are intermediate between those of Δ^9^-THC and Δ^9^-THCP (see next paragraph) (Brown and Harvey 1988). Similar substitution reactions occurred at the same positions as observed for the other two compounds, along with an oxidation of the 11-hydroxyl group to a carboxylic acid. Moreover, they highlighted an additional metabolic pathway leading to poly-hydroxylation on the side chain, a phenomenon not observed before for Δ^9^-THCH and lower homologues (Brown and Harvey 1988).

##### Identification and analysis

Linciano et al. carried out a semi-quantitative determination of the compound in the extract of the FM2 variety by UHPLC-HESI-Orbitrap MS confirming that, due to its low concentration in the plant, such technique is the most suitable for its detection and identification (Linciano et al. 2020a).

##### Recreational use

Marketing strategies for Δ^9^-THCH took place as already described for the other compounds listed in this review. Its discovery attracted the attention of both the consumers looking for new legal substances and the industry that has promptly responded with a range of products spanning tinctures, gummies, and liquid for e-cigarettes. Δ^9^-THCH may be the only active compound or it may be blended in a mixture with other cannabinoids (Binoid 2023).

Sometimes online sales sites propose wide informative pages aimed at presenting the new product, describing the differences and similarities with other cannabinoids, with detailed descriptions of the effects on body and mind (DeltaMunchies 2022; Schmidt 2023). In online forums it tends to be compared with Δ^9^-THC and Δ^9^-THCP in terms of potency: while there is obviously a large amount of subjectivity in the consumers’ experience, it is quite often reported that the psychoactive effect lasts longer than that of Δ^9^-THC (reddit.com. 2023).

#### Δ^9^-Tetrahydrocannabiphorol (Δ^9^-THCP)

(6a*R*,10a*R*)-3-Heptyl-6,6,9-trimethyl-6a,7,8,10a-tetrahydro-6*H*-benzo[*c*]chromen-1-ol.

##### Chemistry and synthesis

Among the about 150 phytocannabinoids produced by *Cannabis sativa* L., (‒)-*trans*-Δ^9^-tetrahydrocannabiphorol (Δ^9^-THCP), first identified by Citti et al. in the FM2 cannabis variety (Citti et al. 2019), has quickly gained great attention from both the pharmaceutical industry and consumers for recreational purposes by virtue of its estimated potency. Hence, being one of the most promising known compounds, it is reasonable to expect, in the medium and long term, the appearance on the market of hemp varieties specially selected in order to increase its content.

Δ^9^-THCP can be stereoselectively synthesized by condensation of 5-heptylbenzene-1,3-diol with (1 *S*,4*R*)-1-methyl-4-(prop-1-en-2-yl)cycloex-2-enol, using *p*TSA as catalyst, for 90 min. Final stereoselective yields are influenced by the duration or the diverse Lewis’ acids (Citti et al. 2019).

##### Pharmacology

Following its discovery, Δ^9^-THCP was immediately studied for its pharmacological effects both in vitro and in vivo. Δ^9^-THCP binds with high affinity to both *h*CB1R and *h*CB2R with a *Ki* of 1.2 and 6.2 nM, respectively. As a result, Δ^9^-THCP in vitro binding affinity resulted 33-fold higher than Δ^9^-THC (*Ki* = 40 nM), 63-fold higher than Δ^9^-tetrahydrocannabivarin (Δ^9^-THCV, *Ki* = 75.4 nM) and 13-fold higher than the recently discovered Δ^9^-THCB (*Ki* = 15 nM) against *h*CB1R (Linciano et al. 2020a; Citti et al. 2019). In addition, Δ^9^-THCP binding affinity for *h*CB2R resulted about 5- to 10-times higher (*Ki* = 6.2 nM) than that of Δ^9^-THC, Δ^9^-THCB, and Δ^9^-THCV, which instead showed a comparable binding affinity with a *Ki*ranging from 36 to 63 nM (Linciano et al. 2020a; Citti et al. 2019).

The cannabimimetic activity in vivo of Δ^9^-THCP was evaluated by the tetrad of behavioural tests on mice, which included the assessment of spontaneous activity, the immobility index (catalepsy), analgesia and changes in rectal temperature. Δ^9^-THCP was found to decrease locomotor activity and rectal temperature, induce catalepsy and produce analgesia miming the properties of a full CB1R agonist using the same dose used for Δ^9^-THC (10 mg/kg). At lower doses, Δ^9^-THCP acted like a partial CB1R agonist like Δ^9^-THC (Citti et al. 2019).

##### Identification and analysis

Considered its low amount in cannabis extracts, HPLC-HRMS is the optimal technology to identify and quantify Δ^9^-THCP as shown in the first study on this compound (Citti et al. 2019).

Another study examined the interference of isomers and related cannabinoids on the Cannabinoids Direct ELISA kit from Immunalysis, as a non-target, similarly structured compound can generate a positive response (Moody et al. 2022). This research demonstrated that structural isomers, such as Δ^8^-THC and Δ^10^-THC, exhibit a higher cross-reactivity than other Δ^9^ counterparts, while Δ^9^-THCP showed minimal cross-reactivity. This brings to the conclusion that despite its pharmacological/psychoactive effects are comparable to Δ^9^-THC even at low doses, the Direct ELISA kit from Immunalysis would not be able to detect Δ^9^-THCP in consumers’ whole blood.

##### Recreational use

Δ^9^-THCP is freely available for purchase on the web. Despite its supposed positive effects are widely highlighted on the label, disclaimers often state that it is not for human consumption but only for collection or technical use. It is sold spiked on plant matrices, in vape cartridges and in edible products like gummies and tinctures (Vivimu 2023; L'ErbaProibita 2023).

Online it is reported by recreational users that effects could last up to 24 h or more but this information is only indicative as the physical characteristics of the consumer are not specified (sex, weight, age, health conditions and habit to use) nor if the product contained only Δ^9^-THCP or other THC related compounds (reddit.com. 2022).

#### Hexahydrocannabutol (HHCB)

(6a*R*,9*R*,10a*R*)-3-Butyl-6,6,9-trimethyl-6a,7,8,9,10,10a-hexahydro-6*H*-benzo[*c*]chromen-1-ol *and* (6a*R*,9*S*,10a*R*)-3-Butyl-6,6,9-trimethyl-6a,7,8,9,10,10a-hexahydro-6*H*-benzo[*c*]chromen-1-ol.

No information is available on the synthesis of hexahydrocannabutol (HHCB), though it can be presumed it is derived from hydrogenation of either Δ^8^- or Δ^9^-THCB, thus through a fully synthetic process. Although no details could be retrieved on its epimeric composition, it can be assumed that the synthetic procedure is adjusted to primarily obtain the (9*R*) epimer, which should be the active one based on the literature reported for standard HHC.

Users on reddit.com report an experience similar to the one tried with hexahydrocannabihexol (HHCH, see next paragraph) when consuming HHCB edibles (reddit.com. 2023). In particular, it has a quick onset of the effects, starting 5 to 15 min after ingestion, to drop just as quickly, thus avoiding the bad side effects generally encompassed the day after (TSM 2023). An informative website reports the effects produced by HHCB including stress reduction, relaxing effect, euphoria, appetite stimulating effect, sleep-inducing effect, and analgesic effect (TSM 2023).

#### Hexahydrocannabihexol (HHCH)

(6a*R*,9*R*,10a*R*)-3-Hexyl-6,6,9-trimethyl-6a,7,8,9,10,10a-hexahydro-6*H*-benzo[*c*]chromen-1-ol *and* (6a*R*,9*S*,10a*R*)-3-Hexyl-6,6,9-trimethyl-6a,7,8,9,10,10a-hexahydro-6*H*-benzo[*c*]chromen-1-ol.

The same considerations drawn for HHCB could apply to HHCH as no information is available in the literature regarding its synthesis and pharmacology. For the same reason, it is presumed that the active epimer is the (9*R*).

Reddit users report different experiences, but the majority say that vaping HHCH distillates gives a mild sensation, even at high concentration levels close to 80%, nothing compared to the corresponding Δ^9^-THC hexyl counterpart Δ^9^-THCH (reddit.com. 2023). On the other hand, HHCH edibles produce a high psychoactive effect (reddit.com. 2023).

HHCH can be found in several products including cookies, gummies, and sprayed herbs (reddit.com. 2023).

#### Hexahydrocannabiphorol (HHCP)

(6a*R*,9*R*,10a*R*)-3-Heptyl-6,6,9-trimethyl-6a,7,8,9,10,10a-hexahydro-6*H*-benzo[*c*]chromen-1-ol *and* (6a*R*,9*S*,10a*R*)-3-Heptyl-6,6,9-trimethyl-6a,7,8,9,10,10a-hexahydro-6*H*-benzo[*c*]chromen-1-ol.

##### Chemistry and synthesis

The most feasible synthesis for “illegal” production of hexahydrocannabiphorol (HHCP) seems to be the one reported by Cornia et al. starting from citronellal and 5-heptylresorcinol (sphaerophorol) instead of olivetol and using diethylaluminum chloride as catalyst (Cornia et al. 1989). However, a semi-synthetic approach has also been attempted by hydrogenation of a *C. sativa* extract previously enriched in Δ^9^-THCP by distillation (Bueno and Greenbaum 2021). More productive and stereoselective synthetic methods would resemble the routes already used in the synthesis of HHC but with an heptyl-substituted resorcinol and (*R*)-citronellal precursors.

##### Pharmacology

A dog ataxia assay of the homologous series of THC and HHC run during the 1940s and in 1950 suggested that the “hexyl-” series is dramatically more potent, THCP is slightly more potent, while all the other compounds (hence including HHCP) resulted of weaker effect compared to Δ^9^-THC (Adams et al. 1941; Loewe 1944; Loewe 1950). These studies were affected by an unknown epimeric purity of the analyte so the proposed ranking should be better considered as indicative.

In vitro tests on the CB1R and CB2R have shown great affinity of both Δ^9^-THCP (Citti et al. 2019) and the metabolite-like 11-OH-9β-HHCP (Ujváry 2023) suggesting that the cannabimimetic effects of HHCP ((9*R*) epimer) would be worth an in-depth evaluation.

##### Identification and analysis

At present, no specific literature is available on analytical methods for the qualitative and quantitative determination of HHCP. On the basis of its chemical structure the same analytical techniques available for other cannabinoids should be fit for the purpose with either GC or HPLC needed to separate the two epimers. Characterization data for this compound (GC-MS, FT-IR, ^1^H NMR, ^13^C APT-NMR, 2D-NMR HSCQ-DEPT, 2D-NMR COSY, 2D-NMR HMBC, 2D-NMR TOCSY, 2D-NMR NOESY were provided to EMCDDA by the Customs Laboratory of Slovenia following the analysis of a seized postal sample (EMCDDA 2023).

##### Recreational use

On the web HHCP is more advertised by purchasers than reviewed by users probably because it is a relatively new compound on the market following research on the boosting effects of the seven carbon atoms chain on previously known cannabinoids. Though comments on its psychoactive effect seldom refer to the use of the single substance, many users report of unprecedented euphoria and in general intense effects but usually uplifting and providing a solid mood boost (Schmidt 2023).

Online retailers claim HHCP has several benefits including strong mental and body experience, anxiolytic properties, relief, happy experience, better mood, great feelings and good times (HHC-P Effects and Benefits: Why Everyone Loves Them; LAWEEKLY 2022). As for other cannabinoids, they provide it in different forms such as tinctures, vape cartridges, gummies (also in combination with other compounds) (binoid 2023).

## Non-natural cannabinoids

### Semi-synthetic non-natural cannabinoids (SSNNCs)

By utilizing Δ^9^-THC or its legal alternative CBD as precursors, new molecules can be synthesized and introduced as purported natural products into the recreational market. Despite originating from natural sources, these compounds are far from being truly natural; rather, they are alterations of well-known cannabinoids such as Δ^9^-THC and HHC. Due to their recent introduction to the market, limited information is available for some of these compounds.

#### Tetrahydrocannabinol acetate (THC-O)

(6a*R*,10a*R*)-6,6,9-Trimethyl-3-pentyl-6a,7,8,10a-tetrahydro-6*H*-benzo[*c*]chromen-1-yl acetate.

##### Chemistry and synthesis

Δ^8^-THC and Δ^9^-THC acetates have been known for a long time (Adams et al. 1945) as final products of the acetylation reaction on Δ^8^-THC and Δ^9^-THC, although the former can be also obtained from the acid-catalysed isomerization of Δ^9^-THC acetate (Gaoni and Mechoulam 1966, 1968).

Following the 2018 Agricultural Improvement Act in the USA, the federal legalization of CBD and its increasing availability on the market worldwide, today the most recurrent, competitive and “legal” synthetic path for cannabinoid acetates involves the acidic catalysed cyclization of hemp derived CBD into a mixture of Δ^8^- and Δ^9^-THC followed by the acetylation step. The ratio of the two THC isomers in the raw mixture, the presence of other minor hemp derived byproducts and of possible contaminants depends on the nature of catalyst, solvent, reaction temperature and purification technology (if any) (EMCDDA 2023; Holt et al. 2022). For this reasons, commercially available products advertised as “THC-O based” in all forms (vape cartridges, edibles, spiked herbal products, etc.), are supposed to contain a variable composition of Δ^8-^ and Δ^9^- acetates rather than an isolated compound, although an extensive analysis campaign has not been conducted yet (Holt et al. 2022).

##### Pharmacology

Being easily associated with the most famous, structurally and name related psychoactive compound of *Cannabis sativa* L. Δ^9^-THC, THC-O has gained great popularity and attention among recreational users of such compounds for being psychoactive itself.

Kruger at al. investigated a social media forum devoted to THC-O-acetate (THCO) highlighting major and minor topics and issues. Among all aspects discussed, the index of the rising interest about THC-O rose from nearly zero participants to almost 6,000 in just one year (Kruger et al. 2023).

Another very recent study relying on surveys to THC-O consumers and addressing specifically its psychedelic action (cognitive distortions such as altered sense of time, weak concentration, failing short-term memory, and hallucinations) reports negative results in this sense, especially among users with prior experience with classic psychedelics (Kruger et al. 2023).

In rhesus monkey behavioural assay, both Δ^8^- and Δ^9^-THC-O administered intravenously at 1 mg/kg elicited similar behavioural changes as those caused by their free derivative (Δ^8^-THC or Δ^9^-THC) at lower doses (0.5–0.9 mg/kg for Δ^8^-THC and 0.1–0.25 mg/kg for Δ^9^-THC, respectively) (Edery et al. 1971, 1972). While Δ^8^-THC showed half the potency of Δ^9^-THC, this difference between the two acetate derivatives could not be appreciated in this in vivo assay (Edery et al. 1971, 1972). Moreover, the onset of effects of the acetates was slightly delayed (15–30 min vs. 10–15 min of the Δ^8^ and Δ^9^phenol isomers) and prolonged (5–6 h vs. 4-4.5 h) suggesting that the acetates act as prodrugs (Edery et al. 1971, 1972). Indeed, with respect to their parent compounds, Δ^8-^ and Δ^9^- acetates show a slight increase in their lipophilicity, which is an advantage in terms of both entering the central nervous system and being protected from metabolic inactivation by conjugation or oxidation. However, being inactive in this form, a subsequent spontaneous or enzymatic hydrolysis delivers the free bioactive phenols (EMCDDA 2023).

As highlighted for cannabinol acetate (CBN-O), Δ^8^-THC-O, CBD diacetate (CBD-di-O) (Munger et al. 2022), and for HHC acetate (HHC-O, see next paragraph), vaping or dabbing products containing Δ^9^-THC-O may expose the consumer to a higher risk of “e-cigarette vaping associated acute lung injury” (EVALI). Indeed, exactly like Vitamine E Acetate (VEA) added to e-liquids, all the cannabinoid acetates have a phenyl acetate moiety, which have been demonstrated to undergo a conversion to phenol and to the highly reactive poisonous gas ketene, the latter a presumptive causative agent of EVALI (Munger et al. 2022).

##### Identification and analysis

Despite being a derivatization product of Δ^9^-THC, THC-O showed a very little cross-reactivity with respect to Δ^9^-THC and its isomers when tested through ELISA test in whole blood (Moody et al. 2022). Hence, present immunoassay technology does not seem to be an effective alternative to more precise, long tested but time-consuming techniques, such as DART-QToF (for exact mass identification), GC-MS and LC-MS/MS for the identification and quantitation (Holt et al. 2022).

##### Recreational use

An in-depth search on one of the most populated online forums among recreational users disclosed controversial effects after taking THC-O: some users report psychedelic-like effects, some others, instead, experienced null or typical THC-like effects. In this regard, the variable product composition may be the root cause for the dissimilar reported effects (Kruger et al. 2023).

On reddit.com users report stronger and delayed effects with respect to Δ^8^-THC and Δ^9^-THC, sometimes with some psychedelic effects like increased time dilation, deeply internalized self-contemplation and complete focus on only one thing at a time (reddit.com. 2021). Apparently, the intensity of these effects tends to diminish quite quickly over time due to tolerance.

#### Hexahydrocannabinol acetate (HHC-O)

(6a*R*,9*R*,10a*R*)-6,6,9-Trimethyl-3-pentyl-6a,7,8,9,10,10a-hexahydro-6*H*-benzo[*c*]chromen-1-yl acetate *and* (6a*R*,9*S*,10a*R*)-6,6,9-Trimethyl-3-pentyl-6a,7,8,9,10,10a-hexahydro-6*H*-benzo[*c*]chromen-1-yl acetate.

##### Chemistry and synthesis

Hexahydrocannabinol acetate (HHCO or HHC-O), has been reported in the scientific literature for the first time in 1942, obtained through catalytic hydrogenation of a mixture of Δ^8^-THC-O and Δ^9^-THC-O in acetic acid and with platinum as catalyst of choice (Wollner et al. 1942). A second common route can also be the acetylation of HHC with acetic anhydride or acetyl chloride similarly to the synthesis of THC acetates (Ujváry 2023). Details on the current manufacturing process on the drug market are not known or available. As for the epimeric composition, it can be assumed that the synthetic route followed leads primarily to the (9*R*) epimer.

##### Pharmacology

The pharmacological profile of HHC-O is still unknown at present, although it could be predicted and investigated starting from studies on THC-like compounds, such as Δ^8^-THC-O and Δ^9^-THC-O, studied both in vitro and in vivo (Ujváry 2023). Indeed, these esters resulted to be typically inactive in vitro as they need a spontaneous or enzymatic hydrolysis in vivo to release the bioactive phenol. In vivo, when tested in several animals, this biotransformation is carried out by hepatic microsomes while in vitro serine hydrolase carboxylesterase was needed as a catalyst (Watanabe et al. 2005).

An additional toxicological aspect linked to the consumption of HHC-O by inhalation and that should be worth additional investigation is its possible role as causative agent of the EVALI. Since ketene has been detected both in model experiments with chemically pure CBN-O, Δ^8^-THC-O and CBD-di-O and in experiments with commercial Δ^8^-THC-O vape pen cartridges, it might also be expected when heating HHC-O (Munger et al. 2022).

##### Identification and analysis

To the authors’ knowledge, no specific analytical method is reported in literature dealing specifically with the identification in HHC-O in any matrix. However, Holt et al. described both GC and LC methods based on direct analysis in real-time–time-of-flight mass spectrometry (DART-ToF-MS) for exact masses identification, GC-MS and HPLC-MS/MS for the identification and quantitation of THC-O and CBD-di-O in some commercial products (Holt et al. 2022). Though unconfirmed these techniques should prove successful also in the determination of HHC-O.

##### Recreational use

In users’ forums HHC-O is indicated as 1.5 times more potent than the original HHC, which, in turn, possesses about the 80% of Δ^9^-THC activity. This makes HHC-O a little stronger than regular Δ^9^-THC (What is HHC-O? The Complete Guide 2022). Users also refer of an array of effects ranging from sedation and relaxation to effects on anxiety and depression, removal of overwhelming thoughts, more relaxed sleep, blissfulness, openness to new sensations and ideas (binoid 2022; Heredia 2022).

Vape cartridges users refer great satisfaction highlighting that it takes a little more than other cannabinoids to produce effects (reddit.com. 2023). What is more, sensations may be weaker after prolonged use. On the contrary, some others refer of panic attacks after consuming edibles with a sort of chronic depressive mood lasting for a few weeks afterward (reddit.com. 2023).

#### 8-Hydroxy-hexahydrocannabinol (8-OH-HHC)

(6a*R*,8*R*,9*S*,10a*R*)-6,6,9-Trimethyl-3-pentyl-6a,7,8,9,10,10a-hexahydro-6*H*-benzo[*c*]chromene-1,8-diol *and* (6a*R*,8*S*,9*R*,10a*R*)-6,6,9-Trimethyl-3-pentyl-6a,7,8,9,10,10a-hexahydro-6*H*-benzo[*c*]chromene-1,8-diol.

##### Chemistry and synthesis

The 8-hydroxy-HHC (8-OH-HHC) can be generally found as a mixture of an equal amount of (8*S*)-OH-(9*R*)-HHC and (8*R*)-OH-(9*S*)-HHC (YLA 2023). All isomers were first synthesized by Mechoulam (Mechoulam et al. 1980). Unlike its regioisomer (10*S*,9*R*)-OH-HHC, this cannabinoid has never been reported to be naturally occurring, but can be rather found as a minor metabolite of HHC (Harvey and Brown 1990). Companies producing and selling 8-OH-HHC ensure a green and metal-free technology starting from hemp derived material, although they do not provide further information (YLA 2023). It is reported to be crystalline, thus physically more stable than distillate, even more than 10-OH-HHC (reddit.com. 2023).

##### Pharmacology

Similar to 10-OH-HHC, this regioisomer was tested by the Mechoulam group who found that the (8*S*,9*R*)- or (8α,9α)- isomer was the active form triggering psychoactive effects in rhesus monkeys (Mechoulam et al. 1980).

##### Identification and analysis

The analysis for identification and quantification can be carried out by GC-MS (YLA 2023; Sams 2023). Harvey and co-workers carried out the GC-MS analysis of the TMS derivatives of HHC stereoisomers and many of their mono-hydroxylated and di-hydroxylated metabolites including 8β-OH-HHC (identified in the paper as 6β-OH-HHC) (Harvey 1981).

##### Recreational use

Users on reddit.com report a quicker onset of the effects compared to HHC but the physical and mental experience is basically the same (reddit.com. 2023).

### Synthetic non-natural cannabinoids (SNNCs)

This section includes both old and newly developed THC-like molecules sharing two characteristics: they cannot be found in nature neither be synthesized from a natural compound.

#### HU-210

(6a*R*,10a*R*)-9-(Hydroxymethyl)-6,6-dimethyl-3-(2-methyloctan-2-yl)-6a,7,10,10a-tetrahydro-6*H*-benzo[*c*]chromen-1-ol.

The synthetic compound known as HU-210, specifically the (‒)-1,1-dimethylheptyl (DMH) homologue of 11-hydroxy-∆^8^-THC, was reportedly discovered in seizures of products labelled as “Spice Gold,” “Spice Silver,” and “Spice Diamond” by the U.S. Customs and Border Protection in 2009 (Coulter et al. 2011). According to the Drug Enforcement Agency, HU-210, along with its non-pharmacologically active enantiomer HU-211 and other SCs, can be found in K2 or Spice, often called “Bliss”, “Black Mamba”, “Bombay Blue”, “Fake Weed”, “Genie”, and “Yucatan Fire” among other aliases (DEA 2019).

##### Chemistry and synthesis

HU-210 was originally synthesized by Mechoulam’s laboratory and belongs to the group of classic cannabinoids, which present the tricyclic benzopyran structure as their skeleton (Mechoulam et al. 1988). The process begins with the esterification with pivalyl chloride of (1*R*,5*S*)-myrtenol, derived from the oxidation of commercial α-pinene. The reduction of the resulting ester with lithium tri-*tert*-butoxyaluminohydride and subsequent condensation with 5-(1,1- dimethylheptyl)-resorcinol lead to the DMH homologue of (3*R*,4*R*)-11-hydroxy-Δ^8^-THC, known as HU-210 (Mechoulam et al. 1988). When the synthesis starts from (1*S*,5*R*)-myrtenol the opposite inactive stereoisomer (3*S*,4*S*)-11-hydroxy-Δ^8^-DMH-THC (HU-211) is obtained (Mechoulam et al. 1988).

##### Pharmacology

The synthetic compound HU-210 exhibits a range of effects at the pharmacological, biochemical and behavioural levels, primarily attributed to its selective agonistic action on CB1R and CB2R, influencing major neurotransmitter systems (Mechoulam et al. 1988; Ottani and Giuliani 2001).

Its pronounced lipophilic nature enables HU 210 to efficiently cross the blood-brain barrier. HU-210 displays significantly higher potency compared to Δ^9^-THC, demonstrating a binding affinity to neuronal CB1R seven times greater both in vitro (Howlett et al. 1990) and in vivo, particularly in mice, rats and pigeons (Mechoulam et al. 1988; Little et al. 1989). The potency ratios between HU-210 and its (+)-isomer HU-211 in the binding affinity to CB1R exceeded 1500, indicating a remarkable level of enantioselectivity and potency, suggestive of specific receptor interactions (Ottani and Giuliani 2001).

HU-210 demonstrates a diverse array of behavioural effects, suggesting alterations in various neurotransmitter systems. Studies in pigeons (Ferrari et al. 1999) and rats revealed HU-210-induced sedation, accompanied by dose-dependent reductions in locomotion, shaking, and rearing (Ferrari et al. 1999;  Martín-Calderón et al. 1998). Moreover, the compound has been found to inhibit hippocampal and medial prefrontal cortex long-term potentiation (Stella et al. 1997). Experiments in rats navigating a water maze task showed time- and dose-dependent interference with learning processes, consistent with observed neurochemical and electrophysiological hippocampal changes (Ottani et al. 2000; Ferrari et al. 1999). However, cannabis-induced impairment of cognitive functioning in human is questionable (Ottani and Giuliani 2001).

Despite inducing marked sedation, high doses of HU-210 in rats led to hypersensitivity to tactile stimuli and robust vocalizations upon touch, reflecting a peculiar blend of depressant and stimulatory effects characteristic of cannabinoids (Ferrari et al. 1999; Ferrari et al. 1999). Vocalization, often associated with cannabimimetic activity, occurred at doses considerably lower than those eliciting similar responses with Δ^9^-THC (Ferrari et al. 1999; Ferrari et al. 1999). This response might suggest heightened emotional states, possibly linked to fear, paralleling anthropomorphic interpretations of aggressive reactions observed in rats administered with HU-210 (Rodríguez de Fonseca et al. 1996), aligning with long-proposed correlations between cannabinoids and stress (Ottani and Giuliani 2001).

Compared to Δ^9^-THC, HU-210 has been found to be substantially more potent (100–500 times) in inducing analgesia and hypothermia in rats (Martín-Calderón et al. 1998), although in CB1R knockout mice Δ^9^-THC, but not HU-210, induced analgesia in the tail flick test (Zimmer et al. 1999).

##### Identification and analysis

Analytical tests on HU-210 are limited due to its frequent presence alongside other substances in small quantities within “Spice”. Additionally, standard drug tests currently fail to detect the use of these particular drugs (Auwärter et al. 2009). However, analysis of*N,O*-bis(trimethylsilyl)acetamide-derivatized and splitless-injected extracts using GC/MS with selected ion monitoring identified HU-210 (DEA 2019).

Another effective method involves utilizing the *Quantisal*device for oral fluid collection, allowing for the simultaneous determination of various “Spice” compounds, including HU-210. This approach has gained the popularity of oral fluid testing in drug detection, particularly in roadside and workplace screenings, due to its convenience, non-invasive nature, and ability to detect recent drug consumption (Coulter et al. 2011).

##### Recreational use

HU-210 is frequently present in the popular “herbal mixture” referred to as “Spice”, a substance commonly smoked for its psychoactive impact, going by names like Genie and Yukatan (Coulter et al. 2011; , Farinha-Ferreira et al. 2022). Users on reddit.com claim higher potency compared to Δ^9^-THC and high safety for this cannabinoid (reddit.com. 2023), while a detailed description of the effects after smoking/vaping is given on bluelight.org (Bluelight 2016). Basically, low doses provide the desired high effects of cannabinoids in very short time, high doses can produce dissociative and psychedelic effects, and lastly medium doses of HU-210 have good analgesic properties (Bluelight 2016). Moreover, all effects are long-lasting (Bluelight 2018).

#### Other new SNNCs

As the scientific research cannot keep up with the recreational experimentation, other SNNCs have already been placed on the market and advertised as legal products claiming a natural origin. However, far from being natural, these products are advertised as new cannabinoids and often listed among the so-called “altnoids” meaning alternative cannabinoids different from Δ^9^-THC and Δ^8^-THC. Specifically, they include several types of THC-like cannabinoids, such as the acetylated derivatives of Δ^9^-THCP (THCP-O), which can be found in blended products with other less common cannabinoids (reddit.com. 2023). Other newly appeared cannabinoids, which can be found in blended products, include THC-JD and THC-X.

All these compounds share the lack of scientific literature on the chemical synthesis and psychoactive effects. On the other hand, the psychonaut websites are rich of information about the effects experienced by recreational users and suggestions on the places where to purchase them.

##### Tetrahydrocannabiphorol acetate (THCP-O)

(6a*R*,10a*R*)-3-Heptyl-6,6,9-trimethyl-6a,7,8,10a-tetrahydro-6*H*-benzo[*c*]chromen-1-yl acetate.

THCP-O is reported to give longer lasting and more euphoric effects compared to Δ^9^-THCP, but also a later onset (up to 30–45 min) (Bluelight 2023), which is in agreement with the metabolization of the acetate prodrug to release the active drug. Moreover, from a chemical-pharmaceutical standpoint, the addition of an acetate group enhances both thermal and oxidative stability, which confers a longer duration of the psychoactive effects (Black et al. 2023). Indeed, the compound has the potential to induce strong sensations of euphoria, relaxation, intoxication, or drowsiness (Canatura 2023). The reported duration of its effects extends up to eight hours, a notably lengthier timeframe compared to the majority of other cannabinoids (Bluelight 2023). Any potential side effects of THCP-O are short-lived and expected to be similar to those associated with other psychoactive cannabinoids, including anxiety, drowsiness, dry mouth, red eyes, and paranoia (Black et al. 2023).

At present, the product range predominantly encompasses e-cigarette liquids and edible gummy lollies products. The majority of these items, alongside THCP-O, incorporate other cannabinoids like THCP or HHCP. Moreover, CBD flowers infused with THCP-O distillate and hashish derived from THCP-O are also available on the market (Canatura 2023).

##### Hexahydrocannabiphorol acetate (HHCP-O)

(6a*R*,9*R*,10a*R*)-3-Heptyl-6,6,9-trimethyl-6a,7,8,9,10,10a-hexahydro-6*H*-benzo[*c*]chromen-1-yl acetate *and* (6a*R*,9*S*,10a*R*)-3-heptyl-6,6,9-trimethyl-6a,7,8,9,10,10a-hexahydro-6*H*-benzo[*c*]chromen-1-yl acetate.

HHCP-O (also known as 9β-hexahydrocannabiphorol acetate, 9β-HHCP acetate, (9*R*)-HHCP acetate or 9β-HHCP-O) (Cayman 2023), is obtained through the hydrogenation of either Δ^8^- or Δ^9^-THCP to become HHCP, followed by acetylation of the OH group (Erickson 2023).

This cannabinoid produces sensations similar to natural cannabinoids like Δ^9^-THC. Despite a lack of scientific articles describing its effects, user experiences shared on blogs and forums indicate that HHCP-O is highly potent, providing strong and long-lasting effects, though it may take some time for the effects to manifest (reddit.com. 2023). Indeed, due to its chemical structure and the presence of an acetate group, HHCP-O ensures effective cannabinoid delivery, albeit with a delayed onset compared to other cannabis concentrates (Thomas 2023). Users report relaxation, intense euphoria, pain relief, warm body vibrations, and even creative inspiration (DottorCanapa 2023). However, it is important to note that even a single use of this compound can potentially lead to withdrawal symptoms (reddit.com. 2023).

HHCP-O is available in various forms, including vape cartridges and dab pens (reddit.com. 2023). The preferred delivery route is the oral consumption, as there is concern about potential risks associated with vaping or dabbing, such as the possibility of EVALI (Munger et al. 2022). The flower containing HHCP-O distillate can be consumed neat or after heating in oil, enabling the creation of edibles while potentially avoiding stomach discomfort (reddit.com. 2023).

##### Tetrahydrocannabinol JD (THC-JD)

(6a*R*,10a*R*)-6,6,9-Trimethyl-3-octyl-6a,7,8,10a-tetrahydro-6*H*-benzo[*c*]chromen-1-ol.

THC-JD is the *n*-octyl synthetic analogue of Δ^8^-THC, which has been reported to have receptor affinity 5-fold greater than that of Δ^8^-THC and pharmacological potencies 10 to 20 times greater than those of Δ^8^-THC (Martin et al. 1999). However, the compound found on the recreational market is not even pure octyl-Δ^8^-THC, but probably a mixture of cannabinoids with only 17% of the octyl-Δ^8^-THC (reddit.com. 2023). Other sources report that THC-JD can be in either the Δ^9^ or Δ^8^form (Johnson 2023). As there is no way to produce THC-JD from CBD as for other cannabinoids, a JD version of CBD should be synthesized first and then converted into THC-JD in the same way as THC is made from CBD (Johnson 2023).

Notwithstanding the great suspicious attitude among the recreational users, someone states that the effects are similar to those of Δ^9^-THC, but with an additional boost, thus enhancing good mood, hunger, sleepiness, and so forth (GasDank 2022). However, the users’ experience are also contradictory sometimes reporting to cause the “couch-lock” effect common in*indica* strains, and other times exhibiting the *sativa*energizing, mood-boosting qualities (GasDank 2022).

##### Tetrahydrocannabinol X (THC-X)

There is significant confusion surrounding the true identity of THC-X, with various websites presenting different interpretations of this cannabinoid. Consensus exists regarding its synthetic nature, but divergent claims include its characterization as a blend of Δ^8^esters (acetoacetate, butyrate, and isovalerate) (TheCalmLeaf 2023; Heidelbaugh 2023), a branding term for Δ^10^-THC (Ward 2024) or even a fluorinated version of Δ^9^-THC with purported pharmacological properties like pain relief, anti-inflammatory effects, and psychoactivity (Delta8Resellers 2023). Despite educational websites providing information, THC-X is promoted as a novel cannabinoid; however, a circulating certificate of analysis has revealed that it is essentially a blended product primarily composed of Δ^8^-THC, Δ^8^-THC-O, Δ^9^-THCP, and small amounts of Δ^9^-THC-O and CBD (SDPharmLabs 2022). The analysis employed HPLC, though the type of detector used was not specified, with a mass spectrometer being a likely possibility.

For recreational users, the sensations claimed are mellowing, relaxing, euphoriant, and energizing, while possible side effects would be anxiety, dry mouth, dry or red eyes, increased appetite, increased heart rate, memory loss, slightly declined cognitive function, and slowed reaction times (Ward 2024).

Online vendors offer diverse THC-X containing products, such as gummies, disposable vape pens and cartridges, tinctures, and flowers (TheCalmLeaf 2023).

## Conclusions

The exceptionally rapid expansion of the recreational cannabinoids market has emerged as a central concern for modern society, posing significant health risks. Despite ongoing rigorous scientific research, conclusive findings on the pharmacological activities, psychoactive effects, and psychological consequences of the use and abuse of these compounds remain elusive. Each time a new cannabinoid is discovered or synthesized, the recreational market is quickly inundated with analogous and more potent derivatives. The scientific community and regulatory institutions struggle to keep pace with this race. Consequently, this review, aiming to encompass as many currently available species in the recreational market as possible, may likely become outdated within a few months. Moreover, the lack of sensitive analytical methods capable of detecting a wide range of molecules, as is currently achieved with cannabinoids for recreational use, prevents users from knowing with certainty the composition of commercial products. Even ingredient claim may turn out to be fake. Consequently, understanding the risks consumers face remains challenging.

The cannabinoids market, in particular, distinguishes itself from other illegal drug markets, as the recreational use of such substances is concealed and somehow justified by the purported beneficial properties of cannabis and hemp. Since many of these compounds originate from CBD, extracted from industrial hemp, they are promoted as legal under the well-known Farm Bill Act issued in 2018 in the USA, arguably the largest market for these products. Furthermore, numerous websites aiming to advertise new products and attract both expert and naïve consumers often provide misleading information about chemical properties, legal status, and potential benefits. Many cannabinoids, such as THCP-O, are promoted as naturally derived from cannabis due to their structural similarity with the well-known THC and the recently discovered THCP. In reality, very few people are aware that a chemical modification, such as acetylation, can profoundly alter the pharmacological properties of a well-known compound and potentially pose unpredictable health risks due to incorrect dosage or an unknown pharmacodynamic and pharmacokinetic profile.

In light of the aforementioned concerns, it is crucial to raise public awareness of the potential risks associated with the use of these compounds. Allocating additional resources to scientific research is imperative to cultivate an expanding knowledge base that can be disseminated widely, reaching diverse audiences, including drug consumers, institutions, and policy makers. To achieve this, a close collaboration between the scientific community and political institutions is essential to ensure successful outcomes for the overall well-being of society and the mitigation of public health risks.

## Data Availability

Not applicable.

## Abbreviations

Altnoids: Alternative cannabinoids
APCI: Atmospheric-pressure chemical ionization
CBC: Cannabichromene
CBD: Cannabidiol
CBE: Cannabielsoin
CBG: Cannabigerol
CBN: Cannabinol
CNS: Central nervous system
CSP: Chiral stationary phase
DAD: Diode array detector
DHC: Dihydrocannabinol
ESI: Electrospray ionization
FID: Flame ionization detector
GC: Gas chromatography
*h*CB1R: Human cannabinoid receptor 1
*h*CB2R: Human cannabinoid receptor 2
HHC: Hexahydrocannabinol
HCl: Hydrochloric acid
HHCA: Hexahydrocannabinolic acid
HHCB: Hexahydrocannabutol
HHCH: Hexahydrocannabihexol
HHC-O: Hexahydrocannabinol acetate
HHCP: Hexahydrocannabiphorol
HHCP-O: Hexahydrocannabiphorol acetate
HMPA: Hexamethylphosphoramide
HPLC: High performance liquid chromatography
HRMS: High-resolution mass spectrometry
IR: Infra-red spectroscopy
MALDI: Matrix-assisted laser/desorption ionization
NMR: Nuclear magnetic resonance
NNCs: Non-natural cannabinoids
OH: Hydroxy
PNCs: Pseudo-natural cannabinoids
*p*TSA: *Para*-toluenesulfonic acid
QToF: Quadrupole-Time of Flight
SAR: Structure-activity relationship
SNNCs: Synthetic non-natural cannabinoids
SPNCs: Synthetic pseudo-natural cannabinoids
SSNNCs: Semi-synthetic non-natural cannabinoids
SSPNCs: Semi-synthetic pseudo-natural cannabinoids
THCH: Tetrahydrocannabihexol
THC: Tetrahydrocannabinol
THCA: Tetrahydrocannabinolic acid
THCB: Tetrahydrocannabutol
THC-O: Tetrahydrocannabinol acetate
THCP: Tetrahydrocannabiphorol
THCP-O: Tetrahydrocannabiphorol acetate
TMS: Trimethylsilyl
